# Deleted copy number variation of Hanwoo and Holstein using next generation sequencing at the population level

**DOI:** 10.1186/1471-2164-15-240

**Published:** 2014-03-27

**Authors:** Dong-Hyun Shin, Hyun-Jeong Lee, Seoae Cho, Hyeon Jeong Kim, Jae Yeon Hwang, Chang-Kyu Lee, JinYoung Jeong, Duhak Yoon, Heebal Kim

**Affiliations:** 1 Department of Agricultural Biotechnology, Animal Biotechnology Major, and Research Institute for Agriculture and Life Sciences, Seoul National University, Seoul 151-921, Korea; 2 Division of Animal Genomics and Bioinformatics, National Institute of Animal science, Rural Development Administration, #564 Omockchun-dong, Suwon 441-706, Korea; 3 Department of Animal Science, Kyungpook National University, Sangju 742-711, Korea; 4 C&K genomics, Seoul National University Mt.4-2, Main Bldg. #514, SNU Research Park, NakSeoungDae, Gwanakgu, Seoul 151-919, Republic of Korea

**Keywords:** Cattle, Hanwoo, Holstein, Copy Number Variation, NGS, Domestication, Selection signal

## Abstract

**Background:**

Copy number variation (CNV), a source of genetic diversity in mammals, has been shown to underlie biological functions related to production traits. Notwithstanding, there have been few studies conducted on CNVs using next generation sequencing at the population level.

**Results:**

Illumina NGS data was obtained for ten Holsteins, a dairy cattle, and 22 Hanwoo, a beef cattle. The sequence data for each of the 32 animals varied from 13.58-fold to almost 20-fold coverage. We detected a total of 6,811 deleted CNVs across the analyzed individuals (average length = 2732.2 bp) corresponding to 0.74% of the cattle genome (18.6 Mbp of variable sequence). By examining the overlap between CNV deletion regions and genes, we selected 30 genes with the highest deletion scores. These genes were found to be related to the nervous system, more specifically with nervous transmission, neuron motion, and neurogenesis. We regarded these genes as having been effected by the domestication process. Further analysis of the CNV genotyping information revealed 94 putative selected CNVs and 954 breed-specific CNVs.

**Conclusions:**

This study provides useful information for assessing the impact of CNVs on cattle traits using NGS at the population level.

## Background

Since the completion of the bovine genome assembly [1-3], a large number of genetic variation as single-nucleotide polymorphisms (SNPs), have become widely known and commercial SNP panels have been developed for cattle [4]. The continued discovery of SNPs in diverse cattle breeds has been further expanded [5,6] by the recent availability of massively parallel sequencing technologies called next-generation sequencing (NGS). SNPs and the commercial SNP marker panels have been successfully used to identify genomic regions that potentially underlie the economic traits of cattle [7-9]. Another source of genetic variation is mammals come from gains and losses of genomic structural sequence variants, copy number variations (CNVs), that occur in more than two individuals [10]. While SNPs are more frequently used in cattle breeding than CNVs, CNVs occupy a higher percentage of genomic sequence than SNPs.

Many studies have endeavored to understand CNVs in mammals, especially in humans [10-13] and rodents [14-17]. In particular, several CNVs were shown to be important in both normal phenotypic variability and disease susceptibility in human [18-22]. It is possible that CNVs have a potentially greater effect on phenotype, including changing of gene structure and dosage, altering gene regulation and exposing recessive alleles [23]. These points are attracting attention to CNV as structural variation that can account for diverse economically important traits in domestic animals. In particular, the CNV type, deletions, which is the focus of this study has been shown to be one of the five CNV types and one of the two main classes with duplications [11]. Previous study of cattle using next generation sequencing (NGS) has reported that CNVs play a crucial role in diverse biological functions as pathogen- and parasite-resistance, lipid transport and metabolism, breed-specific differences in adaptation, health, and production traits [24].

The focus of CNV studies has also extended into other domesticated animals including dog, goat, cattle, pig, and sheep [24-33]. Considering the heritability of CNVs and their higher rates of mutation, CNVs may be largely associated with or affect animal health and production traits under recent selection. In the case of cattle, partial deletion of the bovine gene ED1 causes anhidrotic ectodermal dysplasia [34]. *Bos taurus indicus* has the capacity to adapt to warm climates and superior resistance to tick infestation than *Bos taurus taurus* breeds [35]. Likewise, beef and dairy cattle breeds display distinct patterns in selected metabolic pathways related to muscling, marbling, and milk composition traits. It is possible that CNVs may be associated with these agriculturally important traits [24].

Until now, CNV screens were routinely performed by comparative genomic hybridization (CGH) and SNP arrays, and many studies have extensively reviewed their performances [36-39]. However, these methods, which are often affected by low probe density and cross-hybridization of repetitive sequence, were not able to detect CNVs at the whole genome level. A limited number of investigations in cattle CNV has been performed to detect CNVs using methods that include high-density aCGH and the 50 K SNP panel [25-27]. The recent advances of NGS and complementary analysis programs have provided better approaches to systematically identify CNVs at a deep genome-wide level than the currently available commercial SNP chip and aCGH methodologies [6,40]. These sequence-based approaches, which are becoming more popular due to the ongoing developments and cost decreases in NGS, allow for CNV reconstruction at a higher effective resolution and sensitivity.

In this study, we attempt to detect genome-wide CNVs at the population level based on NGS data of 32 cattle. Using UMD3.1 [3] as a reference genome, we used Genome STRiP to detect cattle CNVs at the population level using Hanwoo (22 individuals), a Korea beef cattle, and Holstein (10 individuals), a dairy cattle. We discovered 18.6 Mbp of deleted sequence in the reference genome. However, using Genome STRiP, we could only extract deleted CNVs from the population data [41]. This study confirmed that CNVs are common, associated with deleted regions, and often occur in gene-rich regions in cattle. We analyzed genes related to CNVs using deletion score in order to explore their potential function and contributions in domestication. In addition, we investigated the selected CNVs using F_ST_ and breed-specific CNVs for traits related to beef and milk production (Additional file 1). By providing several types of information on cattle CNV at the population level and presenting deleted CNV maps with breed-specific CNVs, we provide the basis for further studies into the role of deleted CNVs in the cattle genome.

## Result and discussion

### Result

**Figure 1:**
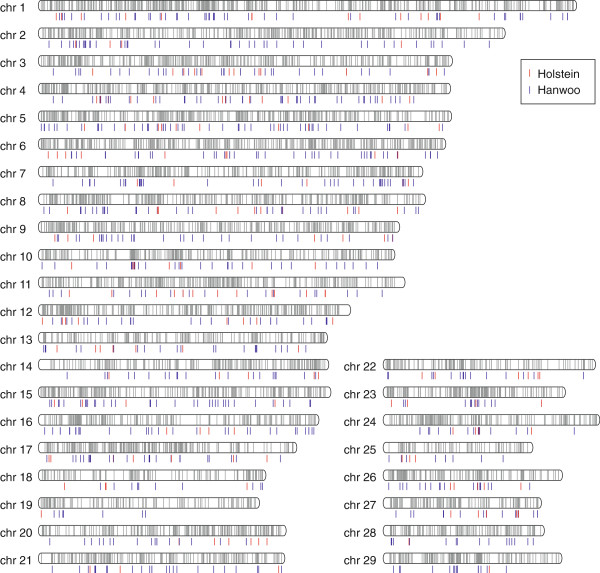
**Cattle deleted CNV map with breed-specific CNVs.** Gray bar represents the cattle deleted CNVs using the entire population of this study. Holstein and Hanwoo-specific CNVs are represented as red and blue bars, respectively. The position of the bars is based on the physical position information of UMD 3.1.

Illumina NGS data were obtained from 10 Holsteins, a dairy cattle, and 22 Hanwoo, a beef cattle. The sequence data for each individual yielded approximately 13.58-fold to 20-fold coverage (Additional file 2). To provide a complete and accurate estimate of CNV at the population level, we used Genome STRiP which combines several technical features including breakpoint-spanning reads, paired-end sequences, and local variation in read depth of coverage [41]. This method had sufficient power to detect deleted CNVs across the autosomes but not enough power to discover inserted events. In this analysis, we focused on the characterization of high-confidence deleted CNVs from known autosomes in UMD 3.1. A total of 6,811 deleted CNVs were detected among the analyzed animals (average length = 2732.2 bp) corresponding to 18.6 Mbp of variable sequence or 0.74% of the entire cattle genome. Using this information, we constructed deleted CNV maps for the cattle genome, which encompassed 1,228 Ensemble cattle reference genes and 2,220 quantitative trait loci (QTL). A full CNV call is shown in the deleted CNV map with breed-specific CNVs (Figure 1). Out of the 6,811 CNVs, 4,407 (9.9 of 18.6 Mbp; 53.1%) were shared between Holstein and Hanwoo and only 2 CNVs (BovineCNV5631, BovineCNV5701) were monomorphic. Information on all CNV regions and individual CNV calls per animal can be found in the CNV information file (Additional file 3).

**Figure 2:**
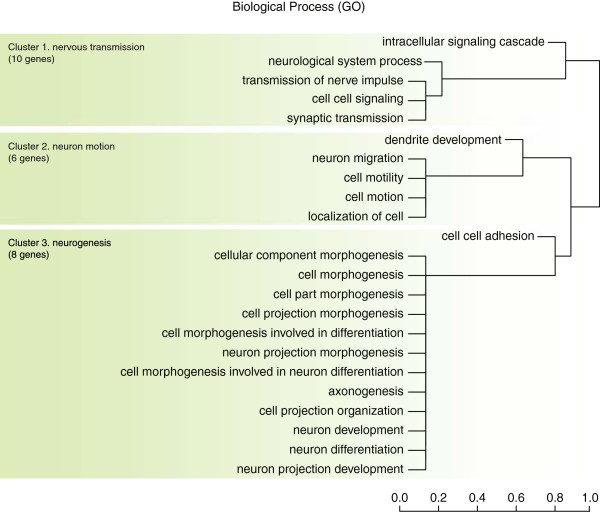
**Hierarchical clustering of significant GO terms for genes with top 1% deletion scores.** Significant results of GO analysis using genes with top 1% deletion scores run with default criteria in DAVID GO analysis (COUNT = 2, EASE = 0.1). These results are largely divided into three clusters: cluster 1 with 10 genes related to nervous transmission. cluster 2 with six genes associated with neuron motion, and cluster 3 with eight genes linked to neurogenesis.

Using the cattle reference gene annotations, we identified CNVs that overlap with genes and then assigned deletion scores to each gene. Out of the 23,431 cattle Ensemble reference genes, 1,228 genes (5.24%) overlapped with the deleted CNVs in this study. The overlapping genes showed high variation in the deletion score with a minimum score of 1, maximum score of 187, median score of 14 and an average score of 21.95. Among the overlapping genes, 33 Ensemble genes had an empirical p-value of less than 0.01 and were considered as being significant in cattle domestication (Additional file 4). While 10 of the 33 Ensemble genes did not have a defined function, Gene Ontology analysis revealed that the remaining 23 genes were related to the nervous system, more specifically nervous transmission, neuron motion, and neurogenesis (Figure 2). Ten genes (cluster 1 of GO analysis, Figure 2) were found to be related to nervous transmission (NCAM2, PIK3C2G, EFNA5, RASGRF2, UNC13C, GUCY1A2, ACCN1, GRM7, DCDC2, and PCDH15). Of these 10 genes, five genes (NCAM2, EFNA5, UNC13C, GRM7, and PCDH15) have been previously reported to be related to nervous transmission [42-49] (Additional file 5). Six genes (cluster 2 of GO analysis, Figure 2) were found to be related to neuron motion (EFNA5, KLHL1, DNAH5, SLIT3, DCDC2, and PRKG1). Five of these genes in cluster 2 (EFNA5, DNAH5, SLIT3, DCDC2, and PRKG1) were reported to be related to neuron motion in previous studies [50-54] (Additional file 5). Eight genes (cluster 3 of GO analysis, Figure 2) were found to be related to neurogenesis (NCAM2, EFNA5, MDGA2, KLHL1, SLIT3, PRKG1, PCDH15, and FAT3). We identified that seven of these eight genes in cluster 3 (NCAM2, EFNA5, MDGA2, KLHL1, SLIT3, PRKG1, FAT3) have previously been reported to be related to neurogenesis [55-61] (Additional file 5). Also, the pathway analysis using 33 significant Ensemble gene IDs based on deletion scores showed that only the pathway related to axon guidance is significant. Three genes, EPHA6, EFNA5, and SLIT3, were associated with this pathway.

QTL related to CNV regions were identified using the Animal QTL database [62]. We found that 2,220 out of 3,605 (61.58%) cattle QTL overlapped with 6,623 putative deleted CNVs. The index used to measure deletion density, the average distance between deletions, showed large variations (minimum: 1069.86 bp; maximum: 3,728,838 bp; median: 20693.06 bp; average score: 31433.17 bp). The top 30 QTL overlapping with CNVs are listed in Additional file 6. CNV deletion scores of the top 30 QTL were also highly variable (between 50 and 142). Six of the top QTL were directly related to meat production while eight of the top QTL were associated with milk production. We also propose genes that overlap with the top 30 QTL (Additional file 6), which are mainly related to sensory perception as olfactory receptor.

**Figure 3:**
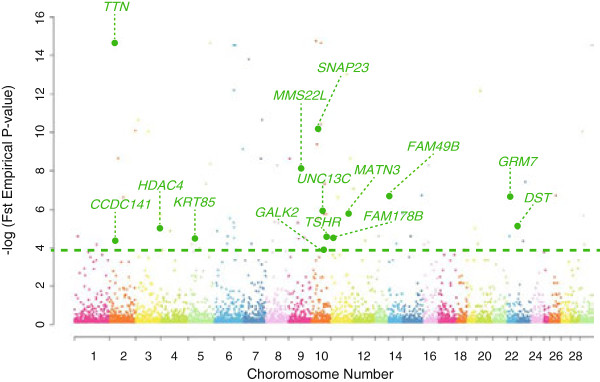
**Manhattan plot of F**_**ST **_**empirical p-value.** Results plotted as negative log-transformed empirical p-values of F_ST_. The green horizontal line indicates the threshold (p-value after FDR correction < = 0.01). The green circle represents the cattle CNVs that are significant and belongs to genic regions.

We identified selective signals between Hanwoo and Holstein populations from CNV based F_ST_ to annotate regions of selection. Differences in the frequencies of deleted CNVs for each breed were used to characterize signatures of selection. The CNVs selected based on F_ST_ exhibited evidence of evolutionary selection in genomic regions that were considered to have been under positive selection in meat and dairy cattle. Ninety-four deleted CNVs were identified as putatively harboring selective sweep signals with FDR multiple test corrected empirical p-values (less than 0.01) of F_ST_ (Additional file 7). Seventeen Ensemble genes overlapped with CNVs and gene function was defined in 14 of the genes (Figure 3). Seven (TTN, MATN3, DST, HDAC4, TSHR, CCDC141, GALK2) of the 14 genes were reported to be related to representative economic traits of each breed [63-68] (Additional file 8). Two (MATN3, DST) of these seven genes had deleted CNVs mainly in Holstein while the other five (TTN, HDAC4, TSHR, CCDC141, GALK2) had deleted CNVs mainly in Hanwoo (Additional file 9).

**Figure 4:**
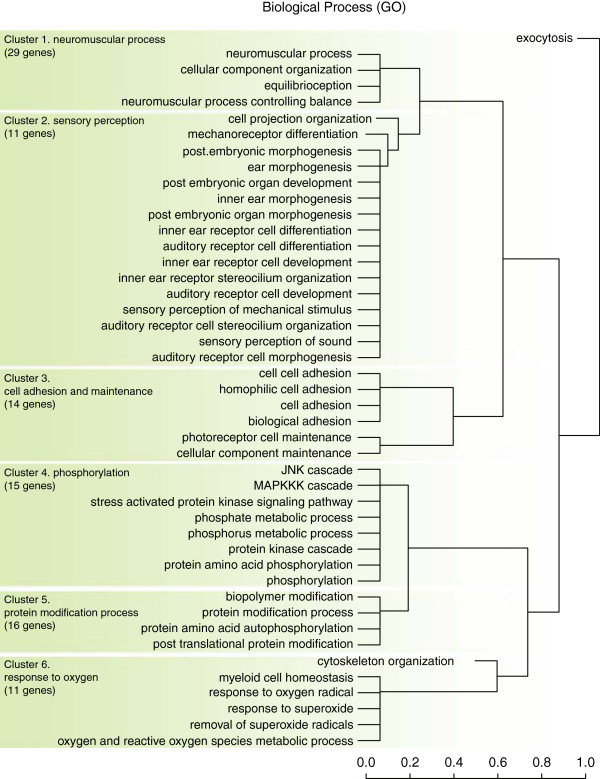
**Hierarchical clustering of significant GO terms for genes that overlap with Hanwoo breed-specific CNVs.** Significant result of GO analysis using genes with top 1% deletion scores run with default criteria in DAVID GO analysis (COUNT = 2, EASE = 0.1). These results are largely divided into six clusters: cluster 1 with 29 genes related to neuromuscular process, cluster 2 with 11 genes associated with sensory perception, cluster 3 with 14 genes linked to cell adhesion and maintenance, cluster 4 with 15 genes related to phosphorylation, cluster 5 with 16 genes associated with protein modification process and cluster 6 with 11 genes linked to response to oxygen and maintenance.

**Figure 5:**
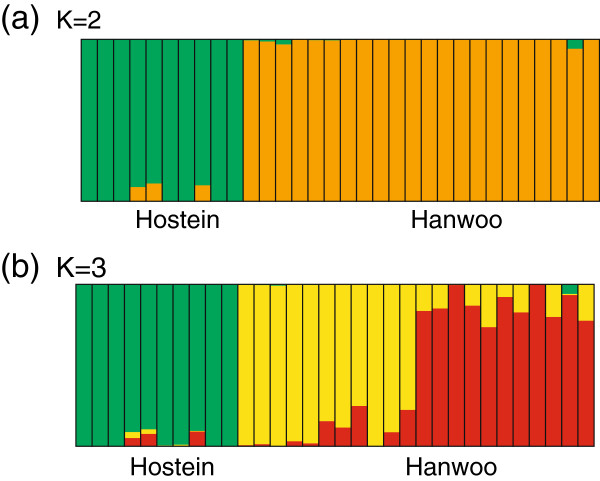
**Population structure analysis using structure.** Each individual is represented by a vertical bar, and the length of each colored segment in each of the vertical bars represents the proportion contributed by ancestral populations. **(a)** Two colors (K = 2) mostly represent population structure of 32 individuals. **(b)** Three colors (K = 3) represent population structure of 32 individuals.

Breed-specific CNVs were identified to investigate their ability to explain breed-specific traits. Although substantial portions of the total CNVs were shared between Holstein and Hanwoo, we found putative breed-specific CNVs for each breed. A total of 2,404 CNVs corresponding to 8.73 Mbp of sequence indicated that deletion was present in only one of the two breeds. After filtering, 767 Hanwoo-specific CNVs and 187 Holstein-specific CNVs were identified (Additional files 10, 11). Hanwoo-specific CNVs were more abundant than Holstein-specific CNVs. We assigned all breed-specific deleted CNVs to a nearby Ensemble gene ID. For the Hanwoo-specific CNVs, 177 of 767 CNVs were related to 173 Ensemble genes, of which 137 had gene symbol for biological interpretation (Additional file 10). Gene Ontology analysis showed that these genes were related to neuromuscular process, sensory perception, cell adhesion and maintenance, phosphorylation, protein modification process, and response to oxygen (Figure 4). Cluster 1 of GO analysis result includes 29 genes (Figure 4) associated with neuromuscular process (ARHGAP10, ARID2, CADPS2, CDH23, CHD9, DNAH9, DSG1, DYNC2H1, EPB41L2, EXOC4, FANCC, GORASP2, ITGAV, KLHL1, LMX1A, MPP6, MYO7A, PALLD, PCDH15, RAPGEF4, RIN3, SMYD3, SOD1, STXBP5L, TLN2, TRPM7, TTF2, USH2A, and UTRN). The second cluster of GO analysis result (Figure 4) includes 11 genes related to sensory perception (CDH23, DNAH9, DYNC2H1, GRM7, KLHL1, LMX1A, MYO7A, NTRK3, PCDH15, SOD1, and USH2A). Cluster 3, which contained 14 genes, was associated with cell adhesion and maintenance (CDH23, CNTN6, COL28A1, DSG1, FAT3, FER, ITGAV, LAMB3, PCDH15, PTPRC, PTPRT, TLN2, TRPM7, and USH2A). Cluster 4 with 15 genes was related to phosphorylation (DAPK1, EPHA5, FER, GAB1, LRRK2, MAP4K3, MAPK10, NDUFA10, NTRK3, PTPRC, PTPRT, RPS6KA2, SOD1, TRPM7, and WNK1) and 16 genes in cluster 5 were linked to protein modification (DAPK1, EPHA5, FBXW2, FER, GAB1, LRRK2, MAP4K3, MAPK10, NTRK3, PTPRC, PTPRT, RPS6KA2, SOD1, TPST1, TRPM7, and WNK1). The final cluster, cluster 6, which includes 11 genes, was connected with response to oxygen (ARHGAP10, CDH23, EPB41L2, FANCC, KLHL1, MYO7A, PALLD, PLCB1, SOD1, TLN2, and TRPM7). Seventeen genes were associated with phosphorylation (cluster 4 in Figure 4) or protein modification process (cluster 5 in Figure 4). We speculate that many of the genes are related to cell growth in phosphorylation and protein modification process, which are needed for the production of meat in the muscle mechanism. Previous studies reported relationships between 10 (NDUFA10, WNK1, MAPK10, FER, RPS6KA2, MAP4K3, PTPRT, PTPRC, GAB1, NTRK3) of the 17 genes related to phosphorylation and protein modification process, and cell growth [69-76] (Additional file 12). Fourteen genes were related to cell adhesion and maintenance (cluster 3 in Figure 5). Out of these, nine genes (ITGAV, COL28A1, FER, TLN2, LAMB3, DSG1, PCDH15, CDH23 and FAT) have been shown to be directly linked to cell adhesion and maintenance [77-85] (Additional file 13). Additionally, it was found that only the neurotrophin signaling pathway was significant when 137 significant genes that overlapped with Hanwoo breed-specific CNVs were analyzed. Five genes, NTRK3, YWHAG, RPS6KA2, GAB1, and MAPK10, were associated with this pathway. For Holstein, 31 out of 187 breed-specific CNVs were related to 26 Ensemble genes (PNKD, PKLR, HCN3, SLC30A7, KIAA1324L, ADCYAP1R1, ZNF804B, NELL2, CNTN1, CRY1, SYNPO2, EFNA5, BAI3, PDE10A, AP3B1, CDAN1, GALM, MATN3, SUGT1, ZMYND8, CUX2, C6ORF10, BRUNOL4, C10ORF28, PSD3, and SLC35F3) (Additional file 11). We predicted that these genes might be linked to dairy production. Previous studies reported relationships between ten (PKLR, ADCYAP1R1, NELL2, CRY1, EFNA5, PDE10A, AP3B1, GALM, MATN3, and C6ORF10) of the 26 genes and dairy production supporting the results of the analysis [64,86-94] (Additional file 14).

**Figure 6:**
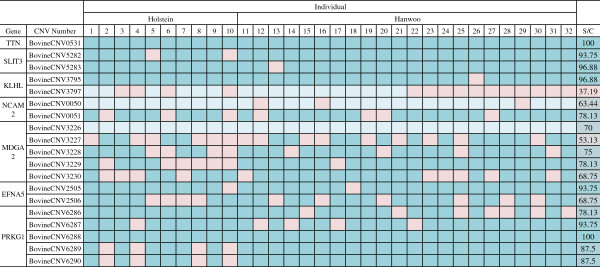
**CNV validation and its accuracy measure by gDNA PCR.** The CNV pattern comparison in 32 individuals was represented by a heat map. The colors of the boxes represent whether the genotypes resulting from PCR matched the predicted genotypes by Genome STRiP. Dark blue indicates that that the two matched while pink indicates that the two did not match. Sky-blue boxes represent restricted matching with the CNV showing only deleted or non-deleted allele in the PCR validation. The average matching rate of the CNVs, 80.02%, was considered to be the CNV accuracy.

To confirm the CNV genotype within some of the putative genes containing the impact of the domestication of cattle, we performed PCR. We selected seven putative genes (TTN, SLIT3, KLHL1, NCAM2, MDGA2, EFNA5 and PRKG1) which had 25 CNVs. However, due to limitations of PCR, we excluded six CNVs that were longer than 1.5 Kb. When the genotype of the examined 19 CNV regions in 10 Holstein and 22 Hanwoo were compared to the expected genotype, various matching rates were discovered (37.19% to 100%, Figure 6, Additional file 15, and Additional file 16). Almost all of the CNV regions examined by PCR showed similar lengths to the expected CNV lengths (< 200 bp) and these CNVs were considered validated. Taken together, the CNV accuracy of this study was determined to be about 80% from the validation experiment (Figure 6).

### Discussion

In this study, we used 32 individual of two cattle breeds, Hanwoo and Holstein, to detect CNVs at the population level. Hanwoo, *Bos taurus coreanae*, is a breed of cattle raised in Korea, which may be a hybrid of *Bos taurus* and *Bos indicus*. Hanwoo migrated and settled in the Korean Peninsula around 5,000 BC. It has been used both as a draft animal and a source of meat but over the past 40 years, the main role of Hanwoo has changed to beef cattle. Since the first official genetic breeding program for Hanwoo by the Korean government started in 1979, the productivity of Hanwoo has improved substantially. In contrast, Holstein is a breed of cattle that has been strongly selected for milk-production and currently has the highest-production of dairy. Genetic resource of Holstein is shared throughout the world by trading in semen and seed bull. We used 22 Hanwoo and 10 Holstein for NGS CNV detection. Holstein individuals were selected using common global criteria while Hanwoo was selected from two different regions to capture the complete genetic picture of the breed. The genetic difference between the Hanwoo individuals of the two populations was identified to be small, and so the 22 individuals were regarded as a single population in this study.

We showed that genes with higher deletion score are more likely to be under genetic drift. Through the CNV deletion score of 32 individuals, we wanted to find out which genes have been affected by cattle domestication. Humans have applied strong selective pressure on each cattle breed through elaborate breeding strategies to form breeds that can provide products such as milk and meat. Animal breeding by humans has been performed during a short period and the cattle population is usually produced by artificial insemination using a small number of seed bull and many cows to both maintain product quality and manage bloodlines. From a genetics point of view, breeding can be regarded as a genetic diversity reduction event much like a population bottleneck. We predicted in this study that deletion regions with beneficial adaptations might have arisen after this genetic diversity reduction event. The loss of variation leaves a surviving population that is favorable with regard to the selective pressures put on it such as the production of milk or meat. The breeding strategies of each cattle breed share a common domestication process, so we wanted to capture the genic regions affected by the general cattle domestication using deleted CNV. Based on this assumption, we selected 33 significant Ensemble genes, which were strongly affected by deleted CNVs using deletion scores. We regarded the genes with higher deletion score as being under neutral or diversifying selection in the absence of additional information.

We want to discover QTL related to the deleted CNVs. We wanted to suggest novel genetic regions over genic regions, which are affected by deleted CNV by using QTL that contain information about the region related to each of the economic phenotype of cattle. So, we used QTL information of Animal QTL to detect a wider region of the genome affected by deleted CNV that contains meaningful information. However, QTL mapping is a step prior to gene definition and QTL region information is roughly defined based on phenotype information. As the variance of QTL length was very large and longer QTL tended to have higher deletion scores (Additional file 17), we could not determine whether high deletion scores of QTL were due to containing many actual deletion regions or simply from the length of the QTL. Therefore, we could not use the deletion scores of QTL to discover QTL affected by deleted CNVs. To overcome this problem, we used a new measurement, average distance between deletions (QTL length/deletion score), to discover QTL affected by deleted CNV. However, the average distance between deletions in QTL was still very variable. It was not possible to create a proper distribution of average distance between deletions in QTL because there were so many QTL regions considering the total number of CNVs. The empirical p-value of QTL had very short average distance between deletions and did not reach the commonly used criteria. However, considering previous studies that discovered important QTL regions that overlapped with SNP in GWAS or selective sweep study, we supposed that QTL containing very short average distance between deletions must be meaningful. So we proposed the top 30 QTL that were selected by the average distance between deletions and regarded these QTL as QTL affected by deleted CNV. We had guessed that QTL types related to CNV in this study would be highly variable, because QTL are roughly defined based on phenotype information and meat and milk traits are complex traits. As expected, the top 30 identified QTL from the QTL analysis had diverse traits. Additionally, as QTL is a region related to economic traits, we focused on the relationship between the region and their traits. We predicted that the gene content of QTL affected by deleted CNV was very important and that this information would supplement information on the genes selected by deletion score.

The domestication and subsequent selection by humans to create breeds have had an impact on the variation within the cattle genome. Strong selection for breed characteristics or productivity has created regions that have lost variation due to the fixation of advantageous mutations, or selective sweep regions. We identified selective sweep regions in the cattle genome but no study has yet to explore these regions using CNVs. In this study, F_ST_ based on the CNV frequency spectra was used to identify and characterize regions of the cattle genome under selective sweep. Additionally, as mentioned earlier, deletion score was used to estimate the genes affected by deleted CNV in cattle domestication by understanding the number of CNVs in each gene and the frequency of each CNV within the population. Selective sweep signal based on F_ST_ of deleted CNV was used to estimate how each deleted CNV affects the trait difference between Holstein (for milk) and Hanwoo (for meat). Between the two examined cattle breeds, 94 putative sweep regions were identified. We assumed that economic traits including beef and milk production have historically been under strong selection. Based on this assumption, we wanted to explore CNVs under selective sweep for economic traits. The results were then used as foundation for the selective sweep section of this study. The most significant deleted CNV (BovineCNV0531) was within the titin gene (TTN). Takahisa Yamada et al. (2009) reported that TTN is involved in myofibrillogenesis through a SNP association study in Japanese Black beef cattle. TTN was reported as the gene which is a positional functional candidate responsible for marbling in beef [63]. A comparison of Japanese Black breed with Holstein and Brown Swiss breed showed that SNP in TTN has strong selection pressure for high marbling [95]. Therefore, even though the deletion was in the intron, we predict that BovineCNV0531 has had strong impacts from selection during breed formation.

Recently, NGS data have been used to discover breed-specific SNP of domesticated animals. In a previous study on pigs, breed-specific SNPs were selected from NGS resequencing data and then filtered by data validation using SNP chip data of many individual to apply assignment test [96]. However, in the case of CNVs it is difficult to validate them, because CNVs in this study are structural variation at the population level that is dependent on the nature of the population and there is no back-up data such as SNP chip data. However, STRUCTURE analysis using 6,811 CNVs could classify individuals into the two breeds, Hanwoo and Holstein (Additional file 18). Therefore, we wanted to know which CNVs were breed-specific and understand the biological meaning of these breed-specific CNVs. We selected CNVs that belonged to only one breed and regarded these CNVs as breed-specific CNV candidates. And then we only selected CNVs with a frequency of higher than 0.1 in each breed to minimize the false positive breed-specific CNV calls instead of validation using back-up data.

If deletions occur within coding regions of the listed genes, the missing functional domains of the translated proteins resulting from that gene may be inferred, we were careful in making such inferences as we did not have the phenotype information that is needed to conduct an association study of the relationship between genetic variants and the traits. Therefore, we could not perform additional analysis or experiment to directly investigate the biological phenomenon affected by deleted CNV. Though there are many limitations, we could discover some key points regarding the missing functional domains of translated proteins resulting from genes largely affected by deleted CNVs.

First, as we only identified deleted CNVs, the results only cover a portion of the genes involved in cattle domestication. In Gene Ontology analysis using the top deletion score genes (23), many genes related to nervous system, more specifically nervous transmission, neuron motion, and neurogenesis were identified (Figure 2). The 23 genes identified as being related to the nervous system may have played a role in the behavioral changes that occurred in cattle due to domestication. During domestication, humans selected for docility in cattle leading to the loss of cattle’s wild nature. Although these genes do not directly code for behavior, they may encode molecular products that govern the functioning of the brain, which then controls character and behavior. A previous study reported that these variations in behavior shape the evolution of genomic elements that influence social behavior through the feedback of selection [97]. The number of genes with a top 1% deletion score was 33 and the number of CNV overlapping with a gene was 135. After comparing the CNV region with the exon region information of 33 genes, only one of 135 overlapped CNVs (BovineCNV3796, chr2: 44486266–44830807) was in exon region. BovineCNV3796 overlapped with 4 exon regions (ENSBTAE00000348420, ENSBTAE00000348416, ENSBTAE00000092579, ENSBTAE00000246220). The remaining 134 CNVs were in intron regions of the 33 genes with a top 1% deletion score We assumed that the extra structure was needed to produce diverse genes during the evolutionary process and CNV affecting these processes remains as an evolutionary trace. So, through the 33 genes with the highest number of CNV deletions, we can observe evolutionary evidence of changes in important cattle character and behavior during domestication by the potential missing functional domains of translated proteins resulting from genes affected by deleted CNVs.

Additionally, we found that 16 protein-coding genes overlapped with the top 30 QTL identified using average distance between deletions. These QTL were largely affected by deleted CNVs. Out of the 16 genes, four (OR10A7, OR2J3, OR6C75, OR6C76) were related to sensory perception as olfactory receptor. Studies of evolutionary changes of a number of ORs in other mammalian species reported that cow has fewer gene in specific OR gene cluster [98]. And a Holstein CNV study reported that there were many CNV losses in several OR genes [99]. The result, which showed that several top QTL overlapped with some of the OR genes, may be supported by these studies. Based on this result, we guessed that OR genes have been affected by domestication process. Moreover, the rearing time for cattle was longer than that of other domesticated mammals. We guessed that the difference among domesticated animals could remain in the OR genes. Previous study of cattle olfactory receptor gene reported that there was significant variation in the genetic component of olfactory receptor systems among artiodactyl species, indicating that the selection pressure for maintaining the integrity of olfactory receptor genes was lower in cattle compared to pigs [100]. These results supported that some CNVs in the selected QTL have been reflected in the evolutionary process during domestication by the missing functional domains of translated proteins resulting from genes affected by deleted CNVs.

In selective sweep signal based on F_ST_ of deleted CNV, 14 protein coding genes overlapped with CNV containing strong selective sweep. Out of these, seven genes (TTN, MATN3, DST, HDAC4, TSHR, CCDC141, and GALK2) were reported as being related to meat or milk production [63-68,101]. In these seven CNVs genotype information, five CNVs had higher deletion frequency in Hanwoo than in Holstein (TTN, HDAC4, TSHR, CCDC141, GALK2) and two CNVs (MATN3, DST) had higher deletion frequency in Holstein. Specially, TTN which encodes an abundant protein of striated muscle is famous as gene related to marbling SNP in Japanese Black beef cattle [63]. We predict that the marbling SNP may give a certain negative effect to muscle production mechanism by TTN gene for intramuscular fat. In this study, all Hanwoo had double deletion CNVs related to TTN genes (Additional file 8). Though CNV extraction in Hanwoo and F_ST_ calculation based on deleted CNV genotype information are trial procedures and not a widely used method, the result matched up with our expectation. Three (HDAV4, TSHR, CCDC141) of other four genes with CNVs that were mainly deleted in Hanwoo were strongly related to muscle (Additional file 9). The last gene, GALK2, has been shown to be up-regulated during the secretory activation in initiation of milk production [68]. In this study, 13 individual had double deletions and 9 individual had single deletions in Hanwoo, but in Holstein only 3 had single deletions and the remaining 7 individuals had no deletions. In the case of CNV that were mainly deleted in Holstein, MATN3 (BovineCNV3277) is related to genetic risk factors for osteoarthritis which is related to dairy production [64]. Nine of the 10 Holstein individuals in this study had more than one deletion, but none of the Hanwoo individuals had deletions. These facts supported that these CNVs have contributed to breed differentiation, perhaps, by missing functional domains of translated proteins resulting from genes affected by deleted CNVs.

In the case of breed-specific CNVs, we selected CNVs in one breed, so there were a higher number of breed-specific CNVs than CNVs found for breed differentiation and it was difficult to discover the biological meaning behind them. In Hanwoo, through GO analysis of genes overlapping with Hanwoo-specific CNVs, two clusters were found. Cluster 1 contains 29 genes and cluster 2 contains 11genes that are related to neuromuscular process and sensory perception, respectively (Figure 4). These terms are similar to genes and QTL strongly affected by deleted CNV. Therefore, we suggest that Hanwoo-specific CNVs reflected the evolutionary process, which occurred during domestication. Additionally, in the case of beef cattle such as Hanwoo, humans have limited the allowed space for the cows to induce better marbling of the meat. Based on these facts, we predict that individuals that are less sensitive may have had more advantages than sensitive individuals in enduring this breeding environment in captivity. Therefore, we supposed that due to this breeding history, genes related to sensory perception and response to oxygen had many deletions. In Holstein, two genes (NELL2, C6ORF10) related to Holstein-specific CNV were down regulated in milk production and one gene (MATN3), related to dairy production, was reported to be a genetic risk factor for osteoarthritis (Additional file 14) [64,88,94]. MATN3 was also selected in the analysis of selective sweep signals based on F_ST_ of deleted CNVs. We predict that Holstein-specific deleted CNV may control some biological process, which gives rise to negative effects on the dairy cattle. These results support the hypothesis that CNVs contribute to breed establishment by the missing functional domains of translated proteins resulting from genes affected by deleted CNVs.

Almost all of the CNV regions examined by PCR in the validation experiment were similar to the CNV regions from Genome STRiP (< 200 bp). However, three CNV regions, BovineCNV 3797, BovineCNV3226 and BovineCNV0050 were not fully validated by PCR assays across both breeds and all surveyed individuals. In BovineCNV 3797 and BovineCNV3226, the deletion alleles were not successfully amplified (case 3 in Additional file 19). This is probably due to the fact that the extracted CNV regions ranged over the primer locations (Additional file 15). Although the deleted allele was not confirmed, the wild-type allele was well defined in this case and in the case of BovineCNV3226, individuals considered to have only a deletion allele did not produce any amplicons. Interestingly, the opposite case, case 4 in Additional file 19, was also present. PCR amplification detected only BovineCNV0050-deleted allele (Additional file 15). So we carried out PCR again using primer pairs amplifying the CNV and its outside region to confirm the presence of the CNV containing allele. However, it did not work and no non-deleted allele was amplified (data not shown). BovineCNV0050 region contains undefined gap sequence, so this could be the reason for the failed amplification. Similar to case 3, the results showing deleted alleles were well defined. When we calculated the CNV accuracy, these two cases were scored lower than case 1 and 2 (0.7 vs. 1.0). The CNV accuracy examined in this study was about 80% (Figure 6).

## Conclusion

Our study presents description of deleted CNVs of cattle by analyzing NGS data of 32 individuals from two breeds. A total of 6,811 deleted CNVs were identified in 22 Hanwoo, and 10 Holsteins individuals. We selected the top 33 genes that had high deletion scores and regarded them as being significantly involved in the domestication process. Their genetic functions were related to nervous system, in particular nervous transmission, neuron motion and neurogenesis. The relationship between these 33 genes and the nervous system may be associated with the changes in behavior due to domestication. The top 30 QTL based on deleted CNVs were associated with diverse quantitative traits including meat and milk production. The genes within top QTL were related to olfactory receptor genes, which reported lower pressure in cattle. We also discovered selective signals in 94 CNVs based on F_ST_ values. The top CNVs that were under selection included the *TTN* gene that has a SNP strongly associated with myofibrillogenesis for marbling in Japanese Black beef cattle. In total, we detected 954 breed-specific CNVs, and 767 of 954 CNVs were Hanwoo-specific and related to several biological processes including phosphorylation, protein modification process, cell adhesion and maintenance, neuromuscular process, sensory perception, and response to oxygen. The other 187 CNVs were Holstein-specific and related to dairy production. Additionally, to confirm the CNV genotype within some putative genes containing the impact on the domestication of cattle, we performed PCR assays. The validation experiment showed that the CNV accuracy of this study is about 80%.

This study provides information on deleted CNVs across the cattle genome at the population level and suggests their possible roles in both domestication and recent breed selection. This study using deleted CNV at the population level is a trial step towards exploring the underlying genetics of economically important traits in cattle and understanding the genetic changes that occurred during domestication. However, further research into the genes related to CNVs and a comprehensive study on inserted CNVs is needed to form a more complete picture of the genetic structure variation in the bovine genome. Additionally, when the associations between CNV and economic traits in cows are identified, it will be possible to incorporate them into breeding programs for production enhancement in cattle.

## Methods

### Ethics statement

All experimental procedures on animals in this study were performed in strict accordance with good animal practice as defined by the relevant national and/or local welfare bodies. In addition, all animal experiments were approved by the Institutional Animal Care and Use Committee of the National Institute of Animal Science (No. 2012-C-005, CNU-00300).

### DNA sampling & resequencing process

Based on the breed history and breed-specific information, we obtained 22 Hanwoo and ten Holsteins for whole-genome resequencing. Individuals were selected as representatives of its breed. Out of the 22 Hanwoo, 11 individuals were from the Hanwoo Experiment Station, National Institute of Animal Science, Rural Development Administration, Korea, and the other 11 individuals were from Kyungpook National University, Korea. Ten Holsteins were obtained from National Institute of Animal Science, Rural Development Administration, Korea. Blood was collected from each animal and treated with heparin to prevent clotting. Manufacturers’ instructions were followed to create a paired library. Pair-end sequence data was generated using Hiseq 2000 (Illumina, Inc). Pair-end sequence reads were mapped to the reference cattle genome UMD 3.1 with aligner based on the Burrows-Wheeler transform and the FM-index (Bowtie2; version 2.1.0) using default setting [102]. Three open-source packages were used for downstream processing and variant calling: Picard Tools, SAMtools, and Genome analysis toolkit (GATK) [103] (Additional file 20). All calls with a Phred-scaled quality of less than 20 were filtered out. The origin, features, and general sequencing information of the individual animals are summarized in Additional file 2.

### Copy number variations extraction

The re-sequencing data of the 32 cows were aligned and CNVs were extracted from the combined dataset. The CNV extraction tool Genome STRucture in Population (Genome STRiP) was used to retrieve deletion calls of CNVs at the population level [41]. Each CNV was genotyped, and the genotype quality was estimated based on the measurement of genotype likelihoods. To ensure that only highly plausible variants are retained, we selected CNVs that passed all genotype quality thresholds in Genome STRiP. Genome STRiP has four filtering criteria for defining deleted CNVs. The definition and default values of the four criteria in Genome STRiP are as follows: COHERENCE (incoherence metric > 0.01), COVERAGE (median normalized read depth of samples with observed evidentiary pairs < 1.0, this filter was used to remove calls in regions of unusually high sequence coverage across many samples), DEPTH (depth ratio < = 0.63 or depth ratio < = 0.8 and heterogeneity P value < 0.01), DEPTHPVAL (Depth p-value using chi-squared test < 0.01). When Genome STRiP defines CNVs, each CNV must pass the four criteria. The number of CNVs decreased from 44,388 to 9,732 CNVs following the filtering criteria. After this step, we applied a secondary criterion to check individual quality for each CNV. In this filtering process, Genome STRiP used genotype likelihoods test. If all individual did not pass through this filtering, we could not obtain the CNV genotype information. After removing the low quality CNVs, 6,811 deleted CNVs remained. We regarded these 6,811 CNVs as the cattle CNVs in this study for additional analyses (Additional file 21).

### Gene content of deleted copy number variations

The gene content of each CNV was assessed by searching each CNV sequence against the Ensemble gene database [104] (http://asia.ensembl.org/index.html). We used BioMart in the Ensemble database to obtain the Ensemble gene IDs for the genes that overlapped with a CNV of the 32 cows [105]. The total number of Ensemble gene IDs was 23,431 and 1,508 CNVs were related to 1,228 Ensemble genes. Deletion score was defined as the number of total deletions in a gene region, as follows:

$$\mathrm{Deletion}\;\mathrm{Score}\;\mathrm{per}\;\mathrm{gene}=\sum_{n=1}^{l}\left(\#\mathrm{Deletion}\;\mathrm{per}\;\mathrm{CNVn}\;\mathrm{in}\;\mathrm{Gene}\right)$$

#Deletion = deletion number in 32 individuals in each CNV (range 0 to 32)

*l =* number of CNVs in each gene

We assigned a deletion score to each gene that overlapped with CNVs (Additional file 22). To discover significant genes that overlap with CNVs that may be affected by the deleted CNV, we calculated empirical p-values for each CNV overlapping gene. We assumed that the distribution of total deletion score values of the 1,228 CNV overlapping genes was a normal distribution. The empirical p-value of each CNV overlapping gene was derived from this normal distribution. Then we selected genes with the top deletion scores (p-value < 0.01) as the representative genes related to cattle domestication. These genes were used to perform Gene Ontology (GO) analysis and pathway analysis in Database for Annotation, Visualization and Integrated Discovery (DAVID; version 6.7) [106].

### Quantitative trait of deleted copy number variations

We compared CNV regions with the cattle QTL regions to explain the role of extracted CNVs in a quantitative trait. The quantitative trait content of each CNV was assessed by selecting QTL regions that overlapped with CNV regions in the 32 cows. The Animal QTL database was used to obtain all QTL region information [62]. QTL traits of cattle can be largely divided into 12 traits with 3,605 loci. The length of QTL was found to be highly variable (minimum: 1000 bp; maximum: 134,956,528 bp; median: 208,803 bp; average score: 7,738,095 bp) (Additional file 23). The average distance between deletions calculated as the QTL length divided by the deletion score was defined as the CNV density within a QTL. The CNV density was calculated for all QTL related to cattle CNV and the top 30 QTL were selected as being representative QTL related to cattle CNV (Additional file 6).

### Population structure analysis & phylogenetic inference

Two preliminary analyses were performed to infer the population structure of the 32 cows used in this study. The program STRUCTURE was used to evaluate the extent of substructuring between Holstein and Hanwoo [107]. We determined that an initial burn-in of 10,000 iterations followed by 10,000 iterations for parameter estimation was sufficient to ensure convergence of parameter estimates. To estimate the number of populations (the K parameter of STRUCTURE), the dataset was analyzed by allowing for the values of K = 2 and 3 (Figure 5 a and b). PCA was conducted for the CNV genotypes in the 32 cows using the statistical program R (Additional file 18). For further identification of the evolutionary history of the samples, we constructed a phylogenetic tree using Bayesian inferences (BI) approaches. Bayesian phylogenetic inference is based on Bayes’s rule. The first characteristic of Bayesian inferences is the use of distribution referred to as the prior that specifies the prior probability of different parameter values. Additionally, this method uses the likelihood function that describes the probability of the data under different parameter values and the total probability of the data summed and integrated over the parameter space to infer a phylogenetic tree. As a result, Bayesian inference is based on the so-called posterior distribution. Phylogenetic analysis in this study was carried out using BI analytical method executed in MrBayes 3.1.2. [108] with the following options: nst: 6, rates: gamma, number of generations: 2,000,000, sample frequency: 100, number of chains: 4, and burn-in generation: 20,000. To estimate the reliability of the nodes, the Bayesian posterior probability (BPP) values were calculated as shown on the BI tree (Additional file 24).

### Identifying selection signal using F_ST_

Wright [109] defined several F coefficients that describe evolutionary processes. His definition was in terms of correlations among gamete: so we used Nei’s equivalent definitions in terms of deviations from expected heterozygosities. $F=\frac{H_{\text{exp}}‒H_{\mathrm{obs}}}{H_{\text{exp}}}$and $\begin{array}{cc} \mathrm{Hobs}=\sum \mathrm{cj}\left(2\mathrm{pjqj}\right) & H_{\text{exp}}=\sum 2E\left(p\right)E\left(q\right) \end{array}$ H_obs_ = the observed frequency of heterozygotes

c_j_ = relative size (proportion) of j^th^ subpopulation

p_j_ = frequency of deletion in j^th^ subpopulation

q_j_ = frequency of allele in jth subpopulation

The estimated values of F_ST_ are shown in Figure 3.

### Identification of breed-specific copy number variations

Each CNV that passed the applied filtering criteria was labeled as a putative breed-specific CNV if the allele was present in only one of the two breeds. Among the putative breed-specific CNVs, CNVs with a deletion frequency of more than 0.1 in each population were selected as breed-specific CNVs. Gene related to the breed-specific CNV were selected and Gene Ontology (GO) analysis was performed in Database for Annotation, Visualization and Integrated Discovery (DAVID; version 6.7) [106].

### CNV validation

We selected seven putative genes (TTN, SLIT3, KLHL1, NCAM2, MDGA2, EFNA5 and PRKG1) that contain the impact of cattle domestication and performed PCR to confirm the 19 CNVs within these genes. Originally, there were 25 CNVs in the seven genes but six CNVs with a length of greater than 1.5 Kb were excluded in this validation. Genomic DNA (gDNA) samples from ten Holstein and 22 Hanwoo were used to validate the CNV region selected by Genome STRiP [41] and determine if they were genuine CNV regions. The primer pairs were designed to be located outside of the predicted CNV region or inside and outside of the CNV region for cases where only the deleted allele was detected (Additional file 20). Fifty nanograms of gDNA was used for PCR amplification and the reaction was performed by using a 2× PCR master mix solution (iNtRON Bio Technology, Seongnam, Gyeonggi, Korea) with 0.5 μM of each primer set. The amplification was performed under the following conditions: 1 cycle of 95°C for 5 min; 35 cycles of 95°C for 30 sec, annealing at the 58 ~ 66°C for 30 sec, and 72°C for 1 min or 1 min 30 sec (Additional file 25); and 1 cycle of 72°C for 10 min. All PCR products were visualized on 1% ethidium bromide stained gels run for 25 min.

### Availability of supporting data

The data set supporting the results of this article is available in the NCBI SRA (PRJNA210523; Hanwoo from RDA in Suwon, PRJNA210521; Holstein from RDA in Suwon, PRJNA210519; Hanwoo from Gyeong-buk).

## Competing interests

The authors declare that they have no competing interests.

## Authors’ contributions

DH carried out the data analyses and interpretation, and mainly drafted the manuscript. HJ (Lee) made substantial contributions to sample acquisition and analysis of genetic data. HJ (Kim) was involved in the drafting of the manuscript and revising it critically for important intellectual content. SA and JY (Jeong) played an important role in performing the statistical analysis and the design of the study. JY (Hwang) and CK participated in the validation experiment. DH and HB conceived of the study, participated in the design and coordination, and helped to draft the manuscript. All authors read and approved the final manuscript.

## Supplementary Material

Additional file 1 Research flow of the study. Click here for file

Additional file 2 Sample information and NGS quality score. Click here for file

Additional file 3 **Total list and genotype information of the cattle deleted CNV.** The genotype information consisted of three types: 0/0, 0/1, 1/1, which indicates non-deletion, single-deletion, and double-deletion, respectively. Sample ID per each branch is in Additional file 1. The CNV name was given by the author in this study. First column includes the CNV name and second column shows the chromosome number of each CNV. Third and fourth columns contain the start and end position of each CNV. The remaining columns represent individuals of Holstein and Hanwoo. Click here for file

Additional file 4 **Deletion score top 1% (p-value < 0.01) genes.** After calculation of deletion score for all genes, we selected the top genes using empirical p-values. These genes were regarded as the representative genes related to cattle domestication. Click here for file

Additional file 5 Deletion score top 1% (p-value < 0.01) genes identified in this study and in previous studies. Click here for file

Additional file 6 **Top 30 QTL using average distance between deletions.** In this study, the average distance between deletions per QTL was used as the deletion density index. After calculating the average distance between deletions for all cattle QTL, we selected the top 30 QTL as being representative of QTL affected by deleted cattle CNV. The QTL names in this study were created by the authors in this study using chromosome and position information. The formal cattle QTL ID from the Animal QTL db are also included. Click here for file

Additional file 7 **Top cattle CNV (p-value after FDR correction < 0.01) using F**_**ST.**_ Click here for file

Additional file 8 **Gene description and reference of the top seven cattle CNVs using F**_**ST**_**, which may impact the differences between Hanwoo and Holstein.** Gene description and references of the top seven cattle CNVs using F_ST_ with their nearby gene identified from this study and previous studies. Click here for file

Additional file 9 **Genotype information of the top seven cattle CNVs using F**_**ST**_**, which may impact the differences between Hanwoo and Holstein.** Genotype information of the top seven cattle CNVs using F_ST_ with their nearby gene identified from this study and previous studies. Click here for file

Additional file 10 Genes that overlapped with Hanwoo breed-specific CNVs. Click here for file

Additional file 11 Genes that overlapped with Holstein breed-specific CNVs. Click here for file

Additional file 12 **Gene description and references for genes related to phosphorylation or protein modification process in Hanwoo.** Gene description and references of some of the genes related to phosphorylation or protein modification process from GO analysis results of genes that overlapped with Hanwoo breed-specific CNV. These genes were identified in both this study and previous studies. Click here for file

Additional file 13 **Gene description and references for genes related to cell adhesion and maintenance in Hanwoo.** Gene description and references of some of the genes related to cell adhesion and maintenance from GO analysis results of genes that overlapped with Hanwoo-specific CNV. These genes were identified in this study and previous studies. Click here for file

Additional file 14 **Gene description and references for genes related to dairy production in Holstein.** Gene description and references of some of the genes related to dairy production. These genes overlapped with Hanwoo breed-specific CNV and were identified in this study and previous studies. Click here for file

Additional file 15 **Genomic DNA amplification in 19 CNV regions.** Genomic DNA from 32 individuals (1–10, Holstein; 11–32, Hanwoo) was used for PCR amplification for validating 19 CNV regions. M indicates a 100 bp-DNA ladder. Arrow and arrowheads show deleted allele and non-deleted allele amplicon, respectively. Predicted length of the PCR products from the deleted and non-deleted alleles are shown in the top left panel of each gel image. Click here for file

Additional file 16 **Genotype comparison between result from genomic DNA amplification and GenomeSTRiP.** The genotype of each CNV and individual are summarized by a heat map. The genotype of the examined 19 CNV regions by PCR was compared to that of the GenomeSTRiP result. Matching score was calculated by dividing the number of individual, whose predicted genotype was the same as the PCR result, to the total number of individuals examined (n = 32). As the CNVs detecting only deleted or non-deleted alleles (BovineCNV0050, BovineCNV3226, and BovineCNV3797) showed limited accuracy, a lower score of 0.7 was used as the matching score. Click here for file

Additional file 17 Relationships between QTL length and QTL deletion score. Click here for file

Additional file 18 **PCA using all deleted cattle CNV as markers.** Green circle represents Holstein and other two colors represent the two different Hanwoo populations. Red diamond represents Hanwoo from RDA in Suwon and yellow represents Hanwoo from Kyungpook National University. Click here for file

Additional file 19 **CNV validation scheme by genomic DNA PCR.** To validate the CNV extracted by GenomeSTRiP, genomic DNA PCR was performed. Primer pairs spanning the extracted CNV were used and each amplicon was visualized by gel electrophoresis. Four patterns of PCR product was detected: the deleted allele being larger or smaller compared to the prediction (case 1 and 2, respectively), absence of deleted allele (case 3) or non-deleted allele (case 4). In case 4, PCR was carried out again with primer pairs which amplify overhanging region between CNV and its outer region. Red and black lines in the diagram representing gel images indicate deleted and non-deleted allele, respectively. Click here for file

Additional file 20 Resequencing NGS data process pipeline before Genome STRiP for CNV extraction. Click here for file

Additional file 21 Distribution per chromosome of the deleted CNV on the cattle genome. Click here for file

Additional file 22 Distribution of the deletion score for the bovine genes. Click here for file

Additional file 23 Histogram of the bovine deleted CNV length. Click here for file

Additional file 24 **Phylogenetic analysis using Bayesian Inference.** Sample ID for each branch is in Additional file 1. Click here for file

Additional file 25 Information of the primer pairs used for zygosity validation. Click here for file
