# Supplementary material for: Deleted copy number variation of Hanwoo and Holstein using next generation sequencing at the population level
Source: BMC Genomics. 2014 Mar 27;15:240. doi: 10.1186/1471-2164-15-240 (PMC4051123; doi:10.1186/1471-2164-15-240)
Supplement: Additional file 1 — Research flow of the study. [file 1471-2164-15-240-S1.PDF]

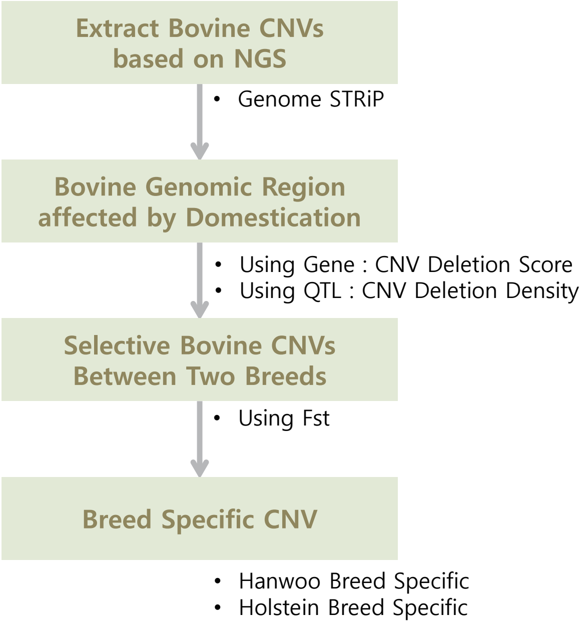
Extract Bovine CNVs based on NGS
• Genome STRiP
Bovine Genomic Region affected by Domestication
• Using Gene : CNV Deletion Score
• Using QTL : CNV Deletion Density
Selective Bovine CNVs Between Two Breeds
• Using Fst
Breed Specific CNV
• Hanwoo Breed Specific
• Holstein Breed Specific
