# Supplementary material for: Deleted copy number variation of Hanwoo and Holstein using next generation sequencing at the population level
Source: BMC Genomics. 2014 Mar 27;15:240. doi: 10.1186/1471-2164-15-240 (PMC4051123; doi:10.1186/1471-2164-15-240)
Supplement: Additional file 2 — Sample information and NGS quality score. [file 1471-2164-15-240-S2.DOCX]

**Additional File 2. Sample information and NGS quality score**

| Sample | Breed | Region | Number of Reads | Align Accuracy | Raw X coverage |
| --- | --- | --- | --- | --- | --- |
| HW_1 | HanWoo | RDA in Suwon | 234,569,860 | 98.04 | 18.68 |
| HW_2 | HanWoo | RDA in Suwon | 187,253,355 | 98.08 | 14.91 |
| HW_3 | HanWoo | RDA in Suwon | 239,492,976 | 98.05 | 19.07 |
| HW_4 | HanWoo | RDA in Suwon | 237,065,133 | 98.08 | 18.87 |
| HW_5 | HanWoo | RDA in Suwon | 217,926,984 | 98.09 | 17.35 |
| HW_6 | HanWoo | RDA in Suwon | 203,717,705 | 98.01 | 16.22 |
| HW_7 | HanWoo | RDA in Suwon | 219,348,590 | 98.18 | 17.46 |
| HW_8 | HanWoo | RDA in Suwon | 212,530,861 | 98.03 | 16.92 |
| HW_9 | HanWoo | RDA in Suwon | 246,479,541 | 97.70 | 19.62 |
| HW_10 | HanWoo | RDA in Suwon | 233,226,278 | 98.05 | 18.57 |
| HW_11 | HanWoo | RDA in Suwon | 217,612,130 | 98.01 | 17.33 |
| HW_12 | HanWoo | Kyungpook National University | 204,631,996 | 97.46 | 16.29 |
| HW_13 | HanWoo | Kyungpook National University | 218,317,802 | 97.34 | 17.38 |
| HW_14 | HanWoo | Kyungpook National University | 214,567,000 | 97.07 | 17.08 |
| HW_15 | HanWoo | Kyungpook National University | 197,016,954 | 97.39 | 15.69 |
| HW_16 | HanWoo | Kyungpook National University | 216,161,654 | 97.41 | 17.21 |
| HW_17 | HanWoo | Kyungpook National University | 213,761,749 | 97.47 | 17.02 |
| HW_18 | HanWoo | Kyungpook National University | 192,688,294 | 96.53 | 15.34 |
| HW_19 | HanWoo | Kyungpook National University | 196,881,793 | 97.32 | 15.67 |
| HW_20 | HanWoo | Kyungpook National University | 176,882,706 | 97.21 | 14.08 |
| HW_21 | HanWoo | Kyungpook National University | 176,265,378 | 97.20 | 14.03 |
| HW_22 | HanWoo | Kyungpook National University | 183,510,511 | 97.27 | 14.61 |
| HS_1 | Holstein | RDA in Suwon | 170,511,249 | 97.31 | 13.58 |
| HS_2 | Holstein | RDA in Suwon | 242,875,799 | 97.35 | 19.34 |
| HS_3 | Holstein | RDA in Suwon | 240,641,118 | 97.48 | 19.16 |
| HS_4 | Holstein | RDA in Suwon | 215,999,610 | 97.15 | 17.20 |
| HS_5 | Holstein | RDA in Suwon | 245,048,441 | 97.08 | 19.51 |
| HS_6 | Holstein | RDA in Suwon | 225,557,863 | 97.77 | 17.96 |
| HS_7 | Holstein | RDA in Suwon | 239,365,182 | 97.45 | 19.06 |
| HS_8 | Holstein | RDA in Suwon | 239,776,485 | 97.17 | 19.09 |
| HS_9 | Holstein | RDA in Suwon | 230,316,320 | 97.75 | 18.34 |
| HS_10 | Holstein | RDA in Suwon | 238,847,676 | 94.42 | 19.02 |
