# Supplementary material for: Deleted copy number variation of Hanwoo and Holstein using next generation sequencing at the population level
Source: BMC Genomics. 2014 Mar 27;15:240. doi: 10.1186/1471-2164-15-240 (PMC4051123; doi:10.1186/1471-2164-15-240)
Supplement: Additional file 4 — Deletion score top 1% (p-value < 0.01) genes. After calculation of deletion score for all genes, we selected the top genes using empirical p-values. These genes were regarded as the representative genes related to cattle domestication. [file 1471-2164-15-240-S4.DOCX]

**Additional File 4. Deletion score top 1 % (p-value < 0.01) genes**

| Ensemble  Gene | Gene Symbol | Chr | Gene Start | Gene End | #CNV | CNV Deletion Score | Empirical P-value |
| --- | --- | --- | --- | --- | --- | --- | --- |
| ENSBTAG00000045905 | PCDH15 | 26 | 5,017,714 | 5,578,654 | 8 | 187 | 2.03E-14 |
| ENSBTAG00000044144 | GUCY1A2 | 15 | 16,604,381 | 17,100,655 | 10 | 177 | 6.20E-13 |
| ENSBTAG00000018996 | BT.61689 | 9 | 98,421,510 | 99,568,077 | 6 | 155 | 5.53E-10 |
| ENSBTAG00000035007 | GALNTL6 | 8 | 3,816,704 | 5,330,369 | 8 | 154 | 7.35E-10 |
| ENSBTAG00000025522 | UNC13C | 10 | 55,717,362 | 56,389,601 | 4 | 143 | 1.48E-08 |
| ENSBTAG00000005697 | MDGA2 | 10 | 39,914,871 | 40,445,335 | 7 | 140 | 3.22E-08 |
| ENSBTAG00000001133 | KIAA0564 | 12 | 11,705,780 | 12,059,605 | 5 | 137 | 6.86E-08 |
| ENSBTAG00000045699 | BT.102825 | 28 | 22,419,203 | 24,270,401 | 5 | 137 | 6.86E-08 |
| ENSBTAG00000013047 | GRM7 | 22 | 18,740,484 | 19,647,747 | 6 | 132 | 2.33E-07 |
| ENSBTAG00000016515 | EFNA5 | 7 | 109,049,590 | 109,217,439 | 4 | 125 | 1.18E-06 |
| ENSBTAG00000002966 | BT.20044 | 1 | 138,139,496 | 138,305,752 | 3 | 105 | 7.14E-05 |
| ENSBTAG00000018404 | PRKG1 | 26 | 6,906,081 | 8,343,629 | 5 | 104 | 8.58E-05 |
| ENSBTAG00000006392 | TTC7B | 10 | 103,185,307 | 103,381,020 | 3 | 99 | 0.00021 |
| ENSBTAG00000008708 | BT.93891 | 12 | 66,292,489 | 67,324,791 | 5 | 97 | 0.00029 |
| ENSBTAG00000025200 | ACCN1 | 19 | 16,353,233 | 17,562,209 | 7 | 93 | 0.00057 |
| ENSBTAG00000027899 | - | 5 | 67,852,917 | 67,930,472 | 3 | 89 | 0.00107 |
| ENSBTAG00000021969 | BT.40893 | 11 | 75,290,644 | 75,623,195 | 3 | 89 | 0.00107 |
| ENSBTAG00000020715 | PIK3C2G | 5 | 91,835,146 | 92,276,939 | 2 | 89 | 0.00107 |
| ENSBTAG00000008647 | KLHL1 | 12 | 44,295,888 | 44,616,940 | 4 | 89 | 0.00107 |
| ENSBTAG00000000655 | MIPOL1 | 21 | 47,852,815 | 48,169,316 | 2 | 84 | 0.00224 |
| ENSBTAG00000009798 | DCDC2 | 23 | 33,102,946 | 33,246,914 | 2 | 84 | 0.00224 |
| ENSBTAG00000021972 | DNAH5 | 20 | 59,285,274 | 59,560,606 | 4 | 84 | 0.00224 |
| ENSBTAG00000005014 | BT.63292 | 11 | 69,204,181 | 69,321,396 | 2 | 83 | 0.00259 |
| ENSBTAG00000004081 | FAT3 | 29 | 1,965,869 | 2,605,125 | 4 | 82 | 0.00298 |
| ENSBTAG00000021291 | BT.88441 | 21 | 35,415,163 | 35,655,955 | 2 | 78 | 0.00513 |
| ENSBTAG00000017746 | SLIT3 | 20 | 324,518 | 507,045 | 2 | 77 | 0.00585 |
| ENSBTAG00000019823 | ADAMTS17 | 21 | 6,514,360 | 6,924,114 | 2 | 76 | 0.00666 |
| ENSBTAG00000030259 | RASGRF2 | 7 | 83,362,788 | 83,546,393 | 3 | 74 | 0.00857 |
| ENSBTAG00000003301 | NCAM2 | 1 | 14,791,090 | 15,026,555 | 2 | 74 | 0.00857 |
| ENSBTAG00000031358 | BT.66173 | 3 | 45,563,732 | 46,487,165 | 4 | 74 | 0.00857 |
| ENSBTAG00000044111 | EPHA6 | 1 | 40,608,729 | 41,623,778 | 3 | 74 | 0.00857 |
| ENSBTAG00000000432 | BT.104278 | 10 | 22,106,909 | 23,304,334 | 2 | 74 | 0.00857 |
| ENSBTAG00000000939 | KIF16B | 13 | 10,238,640 | 10,519,339 | 3 | 73 | 0.00970 |
