# Supplementary material for: Deleted copy number variation of Hanwoo and Holstein using next generation sequencing at the population level
Source: BMC Genomics. 2014 Mar 27;15:240. doi: 10.1186/1471-2164-15-240 (PMC4051123; doi:10.1186/1471-2164-15-240)
Supplement: Additional file 5 — Deletion score top 1% (p-value < 0.01) genes identified in this study and in previous studies. [file 1471-2164-15-240-S5.DOCX]

**Additional File 5. Deletion score top 1 % (p-value < 0.01) genes identified in this study and in previous studies.**

| Clustering Name | Gene | Chr | Reference | Gene Description |
| --- | --- | --- | --- | --- |
| Clustering 1  : nervous transmission | NCAM2 | chr1 | Winther, Berezin et al. 2012 | The protein encoded by NCAM2 may play important roles in selective fasciculation and zone-to-zone projection of the primary olfactory axons. |
|  | EFNA5 | chr7 | McIntyre, Titlow et al. 2010 | EFNA5 Receptors that mediate axonal inhibition or repulsion tended to be expressed in olfactory sensory neurons. |
|  | UNC13C | chr10 | Xu, Wes et al. 1998 | UNC13C encodes protein unc-13 homolog, which has been shown to function in synaptic transmission |
|  | GRM7 | chr22 | Collingridge and Lester 1989,  Meldrum and Garthwaite 1990,  Bliss and Collingridge 1993,  Cartmell and Schoepp 2000 | Metabotropic glutamate receptors of GRM7 are present in varying degree at various synapses and regulate transmitter release.  Glutamate receptors mediate most of the excitatory synaptic transmission in the mammalian central nervous system and play crucial roles in synaptic plasticity, learning and memory, and in some neuropathological disorders. |
|  | PCDH15 | chr26 | Yagi and Takeichi 2000 | Cadherins encoded by PCDH15 have been identified as synaptic components, and their suggested roles include neuronal circuitry, synaptic junction formation, and synaptic plasticity. |
| Clustering2  :neuron motion | EFNA5 | chr7 | Davy, Gale et al. 1999 | Cell surface GPI-bound ligands for Eph receptors are crucial for migration, repulsion and adhesion during neuronal, vascular and epithelial development. |
|  | DNAH5 | chr20 | Sasaki, Shionoya et al. 2000 | This DNAH5 encodes a dynein protein, which is part of a microtubule-associated motor protein complex consisting of heavy, light, and intermediate chains. |
|  | SLIT3 | chr20 | Brose and Tessier-Lavigne 2000 | SLIT3 encodes slit homolog 3, which may act as molecular guidance cue in cellular migration and SLIT proteins were identified as being both negative and positive regulators, repelling various axonal and cell migrations. |
|  | DCDC2 | chr23 | Gleeson, Lin et al. 1999 | DCDC2 encodes a member of the doublecortin family which is a microtubule-associated protein expressed by neuronal precursor cells and immature neurons. |
|  | PRKG1 | chr26 | Bilimoria and Bonni 2013 | The soluble I alpha and I beta isoforms of PRKG by alternative transcript splicing, which play a central role in axon branching. |
| Clustering3  :neurogenesis | NCAM2 | chr1 | Rønn, Hartz et al. 1998 | NCAM have been shown to be crucial for the formation of the olfactory bulb and the mossy fiber system in the hippocampus. |
|  | EFNA5 | chr7 | Hara, Nomura et al. 2010 | Ephrin-A5, a ligand for Eph receptor tyrosine kinases, plays multiple roles in both neurogenesis and vascular formation in the adult hippocampus. |
|  | MDGA2 | chr10 | Litwack, Babey et al. 2004 | MDGA2 protein play a role in neural development, including axon guidance |
|  | KLHL1 | chr12 | Nemes, Benzow et al. 2000 | Protein KLHL1 belongs to a family of actin-organizing proteins related and may play a role in organizing the actin cytoskeleton of the brain cells |
|  | SLIT3 | chr20 | Itoh, Miyabayashi et al. 1998 | The slit proteins may participate in the formation and maintenance of the nervous and endocrine systems by protein–protein interactions |
|  | PRKG1 | chr26 | Yoneyama, Kawada et al. 2011 | The PRKG1 proteins play a role in proliferation of neural stem/progenitor cells |
|  | FAT3 | chr29 | Nagae, Tanoue et al. 2007 | Classic Fat is known to regulate cell proliferation and planar cell polarity And Fat3 plays a role in the interactions between neurites derived from specific subsets of neurons during development. |
