# Supplementary material for: Deleted copy number variation of Hanwoo and Holstein using next generation sequencing at the population level
Source: BMC Genomics. 2014 Mar 27;15:240. doi: 10.1186/1471-2164-15-240 (PMC4051123; doi:10.1186/1471-2164-15-240)
Supplement: Additional file 6 — Top 30 QTL using average distance between deletions. In this study, the average distance between deletions per QTL was used as the deletion density index. After calculating the average distance between deletions for all cattle QTL, we selected the top 30 QTL as being representative of QTL affected by deleted cattle CNV. The QTL names in this study were created by the authors in this study using chromosome and position information. The formal cattle QTL ID from the Animal QTL db are also included. [file 1471-2164-15-240-S6.DOCX]

**Additional File 6. Top 30 QTLs using average distance between deletions**

| QTL Name | QTL ID | Chr | #CNV | #CNV  Deletion Score | QTL Length (bp) | average distance between deletions | QTL Trait | Overlapping Ensemble Gene  (Gene Symbol) |
| --- | --- | --- | --- | --- | --- | --- | --- | --- |
| chr_2_5293683_5505175 | 20298 | 2 | 2 | 82 | 211,492 | 2579.17 | Meat Association | ENSBTAG00000019177 (BT.54746)  ENSBTAG00000045010 (bta-mir-2350)  ENSBTAG00000034949 (HIST1H2AE) |
| chr_3_18099270_18291063 | 13113 | 3 | 2 | 77 | 191,793 | 2490.82 | Health Association  Milk Association | ENSBTAG00000008700 (CRCT1)  ENSBTAG00000045563  ENSBTAG00000046138 (C1ORF68)  ENSBTAG00000032446 |
| chr_4_6695822_6899873 | 1499 | 4 | 2 | 77 | 204,051 | 2650.01 | Health QTL | - |
| chr_5_58828203_59001914 | 15407 | 5 | 5 | 94 | 173,711 | 1847.99 | Reproduction Association | ENSBTAG00000024607 (OR6C75)  ENSBTAG00000046645  ENSBTAG00000006313 (OR6C76)  ENSBTAG00000048224  ENSBTAG00000045684 |
| chr_5_59557792_59731504 | 14027 | 5 | 3 | 81 | 173,712 | 2144.59 | Production Association  Meat Association | ENSBTAG00000039756 (OR10A7)  ENSBTAG00000047259  ENSBTAG00000047967  ENSBTAG00000031097  ENSBTAG00000031096  ENSBTAG00000047619  ENSBTAG00000026078  ENSBTAG00000037629 |
| chr_5_59592534_59766246 | 4412 | 5 | 3 | 81 | 173,712 | 2144.59 | Production QTL | ENSBTAG00000039756 (OR10A7)  ENSBTAG00000047259  ENSBTAG00000047967  ENSBTAG00000031097  ENSBTAG00000031096  ENSBTAG00000047619  ENSBTAG00000037629 |
| chr_5_7933409_8107121 | 20385 | 5 | 2 | 65 | 173,712 | 2672.49 | Meat Association | - |
| chr_5_99090659_99264371 | 5068 | 5 | 3 | 74 | 173,712 | 2347.46 | Reproduction QTL | ENSBTAG00000030468 (BT.76064)  ENSBTAG00000023258 (BT.76070)  ENSBTAG00000030466 (BT.76067)  ENSBTAG00000030461 (BT.76068)  ENSBTAG00000030463 (BT.76065) |
| chr_6_10548494_10723996 | 10146 | 6 | 4 | 92 | 175,502 | 1907.63 | Milk QTL | - |
| chr_6_28262771_28438274 | 16296 | 6 | 3 | 88 | 175,503 | 1994.35 | Health Association | - |
| chr_6_32148739_32324241 | 4541 | 6 | 2 | 66 | 175,502 | 2659.12 | Production QTL | - |
| chr_6_33640512_33816015 | 5382 | 6 | 2 | 110 | 175,503 | 1595.48 | Production QTL | - |
| chr_6_33658063_33833565 | 14705 | 6 | 2 | 110 | 175,502 | 1595.47 | Reproduction Association | - |
| chr_6_92328701_92504203 | 9914 | 6 | 2 | 71 | 175,502 | 2471.86 | Milk QTL | ENSBTAG00000032074 (U1)  ENSBTAG00000007692  ENSBTAG00000017028 (USO1)  ENSBTAG00000015449 (PPEF2) |
| chr_7_31966928_32132336 | 14036 | 7 | 2 | 81 | 165,408 | 2042.07 | Milk Association | ENSBTAG00000020578 (PRDM6)  ENSBTAG00000001568 (PPIC) |
| chr_9_17969953_18152603 | 18466 | 9 | 4 | 78 | 182,650 | 2341.67 | Meat Association | - |
| chr_10_26933288_27109657 | 10052 | 10 | 5 | 91 | 176,369 | 1938.12 | Meat Association | ENSBTAG00000047483  ENSBTAG00000039315  ENSBTAG00000048109  ENSBTAG00000038485  ENSBTAG00000037959  ENSBTAG00000006198  ENSBTAG00000038868  ENSBTAG00000038227  ENSBTAG00000045521  ENSBTAG00000032798 |
| chr_11_99886324_100048919 | 10463 | 11 | 2 | 82 | 162,595 | 1982.87 | Milk Association | ENSBTAG00000019513 (C9ORF50)  ENSBTAG00000015437 (BT.29263) |
| chr_12_49559394_49720836 | 3385 | 12 | 3 | 99 | 161,442 | 1630.73 | Reproduction Association  Production Association | ENSBTAG00000042618 (7SK)  ENSBTAG00000005760 (TBC1D4) |
| chr_12_50520004_50681445 | 5047 | 12 | 3 | 67 | 161,441 | 2409.57 | Milk QTL | - |
| chr_15_3685265_3828842 | 5063 | 15 | 2 | 82 | 143,577 | 1750.94 | Reproduction QTL | - |
| chr_17_23527916_23679836 | 4444 | 17 | 4 | 142 | 151,920 | 1069.86 | Production QTL | - |
| chr_17_24530877_24682797 | 14865 | 17 | 2 | 74 | 151,920 | 2052.97 | Health Association | - |
| chr_18_65270255_65421839 | 6114 | 18 | 3 | 73 | 151,584 | 2076.49 | Milk QTL | - |
| chr_19_7061002_7168138 | 4935 | 19 | 1 | 50 | 107,136 | 2142.72 | Production QTL | - |
| chr_23_20221868_20346736 | 20494 | 23 | 2 | 73 | 124,868 | 1710.52 | Health QTL | ENSBTAG00000021609 (GPR110) |
| chr_23_29418035_29542903 | 12178 | 23 | 4 | 87 | 124,868 | 1435.26 | Meat QTL | ENSBTAG00000038562  ENSBTAG00000040582  ENSBTAG00000027955  ENSBTAG00000040280 (OR2J3)  ENSBTAG00000038928  ENSBTAG00000047558 |
| chr_27_21868197_21976582 | 15284 | 27 | 2 | 53 | 108,385 | 2045.00 | Reproduction Association | - |
| chr_28_10873331_11010357 | 6140 | 28 | 3 | 93 | 137,026 | 1473.40 | Milk QTL | ENSBTAG00000046453 |
| chr_28_17021860_17158886 | 15537 | 28 | 2 | 74 | 137,026 | 1851.70 | Reproduction Association | - |
