# Supplementary material for: Deleted copy number variation of Hanwoo and Holstein using next generation sequencing at the population level
Source: BMC Genomics. 2014 Mar 27;15:240. doi: 10.1186/1471-2164-15-240 (PMC4051123; doi:10.1186/1471-2164-15-240)
Supplement: Additional file 7 — Top cattle CNV (p-value after FDR correction < 0.01) using F ST. [file 1471-2164-15-240-S7.DOCX]

**Additional File 7. Top cattle CNV (p-value after FDR correction < 0.01) using Fst**

| CNV | CHR | Fst | Fst p-value | Fst fdr | Ensemble Gene ID | Gene Symbol |
| --- | --- | --- | --- | --- | --- | --- |
| BovineCNV0531 | chr2 | 0.861 | 2.22E-15 | 2.18E-12 | ENSBTAG00000026986 | TTN |
| BovineCNV1785 | chr5 | 0.730 | 2.22E-15 | 2.18E-12 | - | - |
| BovineCNV2092 | chr6 | 0.727 | 2.89E-15 | 2.18E-12 | - | - |
| BovineCNV2093 | chr6 | 0.727 | 2.89E-15 | 2.18E-12 | - | - |
| BovineCNV2094 | chr6 | 0.727 | 2.89E-15 | 2.18E-12 | - | - |
| BovineCNV2112 | chr6 | 0.727 | 2.89E-15 | 2.18E-12 | - | - |
| BovineCNV3174 | chr10 | 0.733 | 1.67E-15 | 2.18E-12 | - | - |
| BovineCNV3237 | chr10 | 0.730 | 2.22E-15 | 2.18E-12 | - | - |
| BovineCNV6791 | chr29 | 0.727 | 2.89E-15 | 2.18E-12 | - | - |
| BovineCNV2291 | chr7 | 0.709 | 1.52E-14 | 1.04E-11 | - | - |
| BovineCNV3583 | chr11 | 0.689 | 9.36E-14 | 5.80E-11 | ENSBTAG00000021969 | BT.40893 |
| BovineCNV2095 | chr6 | 0.666 | 6.49E-13 | 3.65E-10 | - | - |
| BovineCNV5332 | chr20 | 0.665 | 6.97E-13 | 3.65E-10 | - | - |
| BovineCNV0838 | chr3 | 0.623 | 2.24E-11 | 1.02E-08 | - | - |
| BovineCNV2465 | chr7 | 0.623 | 2.24E-11 | 1.02E-08 | - | - |
| BovineCNV3235 | chr10 | 0.616 | 3.84E-11 | 1.63E-08 | - | - |
| BovineCNV0798 | chr2 | 0.606 | 8.27E-11 | 3.03E-08 | - | - |
| BovineCNV0972 | chr3 | 0.605 | 8.89E-11 | 3.03E-08 | - | - |
| BovineCNV3213 | chr10 | 0.606 | 8.27E-11 | 3.03E-08 | ENSBTAG00000005661 | SNAP23 |
| BovineCNV6673 | chr29 | 0.605 | 8.89E-11 | 3.03E-08 | - | - |
| BovineCNV3187 | chr10 | 0.584 | 4.18E-10 | 1.36E-07 | - | - |
| BovineCNV2212 | chr6 | 0.577 | 7.15E-10 | 2.21E-07 | - | - |
| BovineCNV0573 | chr2 | 0.562 | 2.18E-09 | 6.03E-07 | - | - |
| BovineCNV2450 | chr7 | 0.561 | 2.30E-09 | 6.03E-07 | - | - |
| BovineCNV2826 | chr9 | 0.561 | 2.30E-09 | 6.03E-07 | - | - |
| BovineCNV3623 | chr11 | 0.562 | 2.18E-09 | 6.03E-07 | - | - |
| BovineCNV1786 | chr5 | 0.551 | 4.48E-09 | 1.13E-06 | - | - |
| BovineCNV2663 | chr8 | 0.549 | 5.35E-09 | 1.26E-06 | - | - |
| BovineCNV4652 | chr16 | 0.549 | 5.16E-09 | 1.26E-06 | - | - |
| BovineCNV2969 | chr9 | 0.547 | 6.12E-09 | 1.39E-06 | ENSBTAG00000000629 | MMS22L |
| BovineCNV3817 | chr12 | 0.534 | 1.40E-08 | 3.08E-06 | - | - |
| BovineCNV5921 | chr23 | 0.520 | 3.68E-08 | 7.84E-06 | - | - |
| BovineCNV1728 | chr5 | 0.516 | 4.58E-08 | 9.17E-06 | - | - |
| BovineCNV3287 | chr10 | 0.516 | 4.58E-08 | 9.17E-06 | - | - |
| BovineCNV3665 | chr12 | 0.508 | 8.03E-08 | 1.56E-05 | - | - |
| BovineCNV4561 | chr15 | 0.495 | 1.82E-07 | 3.44E-05 | - | - |
| BovineCNV6325 | chr26 | 0.494 | 1.88E-07 | 3.46E-05 | - | - |
| BovineCNV0642 | chr2 | 0.491 | 2.28E-07 | 3.70E-05 | - | - |
| BovineCNV3463 | chr11 | 0.491 | 2.28E-07 | 3.70E-05 | - | - |
| BovineCNV4137 | chr14 | 0.491 | 2.28E-07 | 3.70E-05 | ENSBTAG00000020801 | FAM49B |
| BovineCNV4138 | chr14 | 0.491 | 2.28E-07 | 3.70E-05 | - | - |
| BovineCNV5731 | chr22 | 0.491 | 2.28E-07 | 3.70E-05 | ENSBTAG00000013047 | GRM7 |
| BovineCNV3268 | chr10 | 0.459 | 1.49E-06 | 0.000230 | ENSBTAG00000025522 | UNC13C |
| BovineCNV4306 | chr14 | 0.459 | 1.49E-06 | 0.000230 | - | - |
| BovineCNV3280 | chr10 | 0.457 | 1.72E-06 | 0.000244 | - | - |
| BovineCNV3313 | chr10 | 0.457 | 1.72E-06 | 0.000244 | - | - |
| BovineCNV3574 | chr11 | 0.457 | 1.72E-06 | 0.000244 | - | - |
| BovineCNV3591 | chr11 | 0.457 | 1.72E-06 | 0.000244 | ENSBTAG00000020893 | MATN3 |
| BovineCNV1122 | chr3 | 0.455 | 1.93E-06 | 0.000268 | ENSBTAG00000000634 | BT.29245 |
| BovineCNV6687 | chr29 | 0.453 | 2.13E-06 | 0.000290 | - | - |
| BovineCNV2042 | chr6 | 0.447 | 2.93E-06 | 0.000384 | - | - |
| BovineCNV3197 | chr10 | 0.447 | 2.93E-06 | 0.000384 | - | - |
| BovineCNV2630 | chr8 | 0.440 | 4.41E-06 | 0.000567 | - | - |
| BovineCNV2091 | chr6 | 0.438 | 4.94E-06 | 0.000601 | - | - |
| BovineCNV2677 | chr8 | 0.438 | 4.94E-06 | 0.000601 | - | - |
| BovineCNV2936 | chr9 | 0.438 | 4.94E-06 | 0.000601 | - | - |
| BovineCNV2097 | chr6 | 0.427 | 8.72E-06 | 0.001042 | - | - |
| BovineCNV5258 | chr19 | 0.426 | 9.11E-06 | 0.001052 | - | - |
| BovineCNV5823 | chr23 | 0.426 | 9.11E-06 | 0.001052 | ENSBTAG00000021237 | DST |
| BovineCNV1125 | chr3 | 0.423 | 1.09E-05 | 0.001233 | ENSBTAG00000017764 | HDAC4 |
| BovineCNV1257 | chr4 | 0.419 | 1.29E-05 | 0.001421 | - | - |
| BovineCNV1876 | chr6 | 0.419 | 1.29E-05 | 0.001421 | - | - |
| BovineCNV0045 | chr1 | 0.407 | 2.38E-05 | 0.002421 | - | - |
| BovineCNV2014 | chr6 | 0.407 | 2.38E-05 | 0.002421 | - | - |
| BovineCNV3997 | chr13 | 0.407 | 2.38E-05 | 0.002421 | - | - |
| BovineCNV4415 | chr15 | 0.407 | 2.38E-05 | 0.002421 | - | - |
| BovineCNV5714 | chr22 | 0.407 | 2.38E-05 | 0.002421 | - | - |
| BovineCNV3339 | chr10 | 0.405 | 2.68E-05 | 0.002688 | ENSBTAG00000017489 | TSHR |
| BovineCNV2096 | chr6 | 0.403 | 2.97E-05 | 0.002848 | - | - |
| BovineCNV3021 | chr9 | 0.403 | 2.97E-05 | 0.002848 | - | - |
| BovineCNV3377 | chr11 | 0.403 | 2.97E-05 | 0.002848 | ENSBTAG00000006019 | FAM178B |
| BovineCNV1583 | chr5 | 0.401 | 3.27E-05 | 0.003049 | ENSBTAG00000026880 | KRT85 |
| BovineCNV2946 | chr9 | 0.401 | 3.27E-05 | 0.003049 | - | - |
| BovineCNV0527 | chr2 | 0.394 | 4.55E-05 | 0.003972 | ENSBTAG00000027875 | CCDC141 |
| BovineCNV2602 | chr8 | 0.395 | 4.38E-05 | 0.003972 | ENSBTAG00000023143 | - |
| BovineCNV3820 | chr12 | 0.395 | 4.38E-05 | 0.003972 | - | - |
| BovineCNV5894 | chr23 | 0.394 | 4.55E-05 | 0.003972 | - | - |
| BovineCNV5895 | chr23 | 0.394 | 4.55E-05 | 0.003972 | - | - |
| BovineCNV4648 | chr16 | 0.392 | 5.06E-05 | 0.004366 | - | - |
| BovineCNV2007 | chr6 | 0.388 | 6.19E-05 | 0.005078 | - | - |
| BovineCNV2326 | chr7 | 0.388 | 6.19E-05 | 0.005078 | - | - |
| BovineCNV4229 | chr14 | 0.388 | 6.19E-05 | 0.005078 | - | - |
| BovineCNV5300 | chr20 | 0.388 | 6.19E-05 | 0.005078 | - | - |
| BovineCNV0292 | chr1 | 0.387 | 6.57E-05 | 0.005147 | - | - |
| BovineCNV1541 | chr5 | 0.387 | 6.57E-05 | 0.005147 | - | - |
| BovineCNV4595 | chr16 | 0.387 | 6.57E-05 | 0.005147 | - | - |
| BovineCNV6696 | chr29 | 0.387 | 6.57E-05 | 0.005147 | - | - |
| BovineCNV1640 | chr5 | 0.377 | 0.000105 | 0.008117 | - | - |
| BovineCNV1152 | chr4 | 0.371 | 0.000135 | 0.009777 | - | - |
| BovineCNV1768 | chr5 | 0.371 | 0.000135 | 0.009777 | - | - |
| BovineCNV2124 | chr6 | 0.371 | 0.000135 | 0.009777 | - | - |
| BovineCNV3277 | chr10 | 0.371 | 0.000135 | 0.009777 | ENSBTAG00000004011 | GALK2 |
| BovineCNV4633 | chr16 | 0.371 | 0.000135 | 0.009777 | - | - |
| BovineCNV5121 | chr18 | 0.371 | 0.000135 | 0.009777 | - | - |
