# Supplementary material for: Deleted copy number variation of Hanwoo and Holstein using next generation sequencing at the population level
Source: BMC Genomics. 2014 Mar 27;15:240. doi: 10.1186/1471-2164-15-240 (PMC4051123; doi:10.1186/1471-2164-15-240)
Supplement: Additional file 8 — Gene description and reference of the top seven cattle CNVs using F ST , which may impact the differences between Hanwoo and Holstein. Gene description and references of the top seven cattle CNVs using FST with their nearby gene identified from this study and previous studies. [file 1471-2164-15-240-S8.DOCX]

**Additional File 9. Gene description and reference of the top seven cattle CNVs using Fst, which may impact the differences between Hanwoo and Holstein**

| CNV | Gene | Chr | Reference | Gene Description |
| --- | --- | --- | --- | --- |
| BovineCNV0531 | TTN | chr2 | Yamada, Sasaki et al. 2009 | TTN is involved in myofibrillogenesis and through association study of a single nucleotide polymorphism (SNP) in Japanese Black beef cattle. |
| BovineCNV3591 | MATN3 | chr11 | Yucesoy, Charles et al. 2013 | MATN3 is related to genetic risk factors for osteoarthritis which related to dairy production. |
| BovineCNV5823 | DST | chr23 | Cole, Wiggans et al. 2011 | The second most significant SNP effect in US holstein GWAS study for daughter stillbirth was the dystonin gene (DST) on BTA23. |
| BovineCNV1125 | HDAC4 | chr3 | Youn, Grozinger et al. 2000 | HDAC4 constitute a family of calcium-sensitive transcriptional repressors of myocyte enhancer factor 2. |
| BovineCNV3339 | TSHR | chr10 | Pipes, Bauman et al. 1963 | TSHR encodes thyroid stimulating hormone receptor and it has been shown that in beef cattle there is a significantly lower thyroxine secretion rate than in dairy cattle. |
| BovineCNV0527 | CCDC141 | chr2 | Fukuda, Sugita et al. 2010 | CCDC141 encodes Coiled-Coil Protein Associated With Myosin II. |
| BovineCNV3277 | GALK2 | chr10 | Mohammad, Hadsell et al. 2012 | GALK2 has been shown to be upregulated during the secretory activation in initiation of milk production. |
