# Supplementary material for: Deleted copy number variation of Hanwoo and Holstein using next generation sequencing at the population level
Source: BMC Genomics. 2014 Mar 27;15:240. doi: 10.1186/1471-2164-15-240 (PMC4051123; doi:10.1186/1471-2164-15-240)
Supplement: Additional file 10 — Genes that overlapped with Hanwoo breed-specific CNVs. [file 1471-2164-15-240-S10.DOCX]

**Additional File 10. Genes that overlapped with Hanwoo breed-specific CNVs**

| Ensemble Gene | Gene Symbol | Chr | Gene Start | Gene End | # Breed  Specific CNV | # CNV |
| --- | --- | --- | --- | --- | --- | --- |
| ENSBTAG00000021396 | HEG1 | 1 | 70,027,330 | 70,106,415 | 1 | 2 |
| ENSBTAG00000021703 | MED12L | 1 | 117,548,538 | 117,917,463 | 2 | 2 |
| ENSBTAG00000030913 | MX1 | 1 | 143,176,083 | 143,204,865 | 1 | 1 |
| ENSBTAG00000020990 | P2RY14 | 1 | 117,785,180 | 117,817,225 | 1 | 1 |
| ENSBTAG00000001918 | STXBP5L | 1 | 66,213,612 | 66,537,723 | 1 | 1 |
| ENSBTAG00000004891 | OXNAD1 | 1 | 155,017,757 | 155,158,936 | 1 | 1 |
| ENSBTAG00000013663 | C1H3orf26 | 1 | 43,730,028 | 44,124,279 | 1 | 1 |
| ENSBTAG00000026994 | C2H2orf88 | 2 | 6,038,403 | 6,113,008 | 1 | 2 |
| ENSBTAG00000044009 | PPP1R1C | 2 | 14,502,890 | 14,623,643 | 1 | 2 |
| ENSBTAG00000020984 | RAPGEF4 | 2 | 23,644,876 | 23,974,945 | 1 | 1 |
| ENSBTAG00000013218 | GORASP2 | 2 | 25,566,351 | 25,599,241 | 1 | 1 |
| ENSBTAG00000011649 | FARSB | 2 | 111,504,465 | 111,578,408 | 1 | 1 |
| ENSBTAG00000012217 | PLA2G2F | 2 | 133,111,283 | 133,124,161 | 1 | 1 |
| ENSBTAG00000023963 | RHBDD1 | 2 | 115,826,528 | 115,960,780 | 1 | 1 |
| ENSBTAG00000019929 | ITGAV | 2 | 9,651,631 | 9,760,100 | 1 | 1 |
| ENSBTAG00000000937 | SSFA2 | 2 | 14,683,868 | 14,751,322 | 1 | 1 |
| ENSBTAG00000025621 | HNRNPA1 | 3 | 54,445,872 | 54,446,858 | 4 | 4 |
| ENSBTAG00000012025 | LMX1A | 3 | 3,692,343 | 3,865,786 | 1 | 3 |
| ENSBTAG00000043876 | U2 | 3 | 54,610,767 | 54,610,931 | 2 | 2 |
| ENSBTAG00000015392 | TTF2 | 3 | 26,244,074 | 26,297,632 | 1 | 1 |
| ENSBTAG00000003279 | NDUFA10 | 3 | 119,779,215 | 119,794,481 | 1 | 1 |
| ENSBTAG00000005439 | FAM102B | 3 | 34,839,059 | 34,877,721 | 1 | 1 |
| ENSBTAG00000046773 | MCOLN2 | 3 | 59,303,476 | 59,351,124 | 1 | 1 |
| ENSBTAG00000032121 | C7orf10 | 4 | 80,866,977 | 81,642,046 | 2 | 3 |
| ENSBTAG00000010437 | MKLN1 | 4 | 95,807,761 | 96,016,756 | 1 | 2 |
| ENSBTAG00000014112 | EXOC4 | 4 | 97,791,626 | 98,594,890 | 1 | 3 |
| ENSBTAG00000024420 | COL28A1 | 4 | 15,332,616 | 15,511,692 | 1 | 1 |
| ENSBTAG00000005110 | CADPS2 | 4 | 87,522,151 | 88,100,974 | 1 | 4 |
| ENSBTAG00000015303 | MPP6 | 4 | 71,625,584 | 71,690,219 | 1 | 1 |
| ENSBTAG00000032650 | DPY19L2 | 4 | 62,259,290 | 62,349,341 | 1 | 1 |
| ENSBTAG00000042539 | U6 | 4 | 47,483,218 | 47,483,324 | 1 | 1 |
| ENSBTAG00000032148 | TMEM117 | 5 | 36,197,272 | 36,807,308 | 2 | 4 |
| ENSBTAG00000026880 | KRT85 | 5 | 27,711,064 | 27,729,751 | 1 | 2 |
| ENSBTAG00000027064 | BTBD11 | 5 | 70,923,456 | 71,257,389 | 1 | 2 |
| ENSBTAG00000021287 | SLC16A7 | 5 | 53,987,909 | 54,214,799 | 1 | 1 |
| ENSBTAG00000013333 | GYS2 | 5 | 89,020,801 | 89,077,024 | 1 | 1 |
| ENSBTAG00000011087 | ARID2 | 5 | 34,488,354 | 34,583,315 | 1 | 1 |
| ENSBTAG00000020914 | CPNE8 | 5 | 42,421,554 | 42,698,411 | 1 | 1 |
| ENSBTAG00000016274 | CCDC38 | 5 | 60,564,497 | 60,591,379 | 1 | 1 |
| ENSBTAG00000004569 | GLIPR1L1 | 5 | 4,824,769 | 4,866,969 | 1 | 1 |
| ENSBTAG00000016260 | LRRK2 | 5 | 40,703,505 | 40,916,225 | 1 | 1 |
| ENSBTAG00000016204 | C1RL | 5 | 103,633,960 | 103,644,323 | 1 | 1 |
| ENSBTAG00000008595 | PPHLN1 | 5 | 38,397,360 | 38,571,901 | 1 | 1 |
| ENSBTAG00000013912 | TXNRD1 | 5 | 68,239,611 | 68,302,678 | 1 | 1 |
| ENSBTAG00000005221 | WNK1 | 5 | 108,079,510 | 108,207,083 | 1 | 1 |
| ENSBTAG00000006156 | BST1 | 6 | 115,687,877 | 115,716,694 | 1 | 2 |
| ENSBTAG00000035776 | C6H4orf22 | 6 | 96,794,894 | 97,503,220 | 1 | 3 |
| ENSBTAG00000009438 | EPHA5 | 6 | 82,560,093 | 82,962,887 | 1 | 1 |
| ENSBTAG00000020048 | MAPK10 | 6 | 102,687,307 | 103,063,481 | 1 | 1 |
| ENSBTAG00000002348 | SLC4A4 | 6 | 88,182,303 | 88,541,046 | 1 | 1 |
| ENSBTAG00000039275 | ERAP2 | 7 | 98,715,725 | 98,767,729 | 1 | 2 |
| ENSBTAG00000009975 | PBX4 | 7 | 3,631,055 | 3,685,738 | 1 | 2 |
| ENSBTAG00000038117 | MGC138057 | 7 | 84,242,450 | 84,505,756 | 1 | 1 |
| ENSBTAG00000003051 | FER | 7 | 110,465,382 | 110,864,228 | 1 | 1 |
| ENSBTAG00000030210 | SLCO6A1 | 7 | 103,818,199 | 103,932,551 | 1 | 2 |
| ENSBTAG00000014661 | CHSY3 | 7 | 25,178,592 | 25,466,387 | 1 | 1 |
| ENSBTAG00000013810 | GABBR2 | 8 | 63,840,064 | 64,089,259 | 1 | 2 |
| ENSBTAG00000000738 | DAPK1 | 8 | 82,207,240 | 82,309,507 | 1 | 1 |
| ENSBTAG00000017195 | FANCC | 8 | 83,023,629 | 83,270,596 | 1 | 2 |
| ENSBTAG00000000712 | FBXW2 | 8 | 112,057,391 | 112,089,639 | 1 | 1 |
| ENSBTAG00000005247 | FRMD3 | 8 | 77,785,287 | 77,910,313 | 1 | 1 |
| ENSBTAG00000001081 | PALLD | 8 | 571,565 | 923,258 | 1 | 1 |
| ENSBTAG00000008836 | ZNF782 | 8 | 84,850,159 | 84,937,203 | 1 | 1 |
| ENSBTAG00000021741 | RPS6KA2 | 9 | 102,918,982 | 103,074,109 | 1 | 1 |
| ENSBTAG00000018634 | SH3BGRL2 | 9 | 19,526,852 | 19,547,939 | 1 | 1 |
| ENSBTAG00000005960 | EPB41L2 | 9 | 69,916,605 | 70,027,346 | 1 | 1 |
| ENSBTAG00000009665 | UTRN | 9 | 82,762,003 | 83,311,756 | 1 | 2 |
| ENSBTAG00000031165 | TRPM7 | 10 | 59,853,461 | 59,943,298 | 1 | 1 |
| ENSBTAG00000003667 | TLN2 | 10 | 47,303,876 | 47,796,327 | 1 | 1 |
| ENSBTAG00000021876 | WDR72 | 10 | 56,678,901 | 56,805,084 | 1 | 1 |
| ENSBTAG00000018947 | SYT16 | 10 | 74,384,705 | 74,497,702 | 1 | 1 |
| ENSBTAG00000008466 | CCNB1IP1 | 10 | 26,830,647 | 26,838,482 | 1 | 1 |
| ENSBTAG00000025642 | RYR3 | 10 | 28,789,149 | 29,100,901 | 1 | 1 |
| ENSBTAG00000044173 | EHBP1 | 11 | 61,117,376 | 61,472,058 | 1 | 1 |
| ENSBTAG00000016442 | MAP4K3 | 11 | 21,579,432 | 21,761,348 | 1 | 1 |
| ENSBTAG00000008647 | KLHL1 | 12 | 44,295,888 | 44,616,940 | 1 | 4 |
| ENSBTAG00000004165 | CLYBL | 12 | 80,409,751 | 80,638,113 | 1 | 2 |
| ENSBTAG00000000939 | KIF16B | 13 | 10,238,640 | 10,519,339 | 1 | 3 |
| ENSBTAG00000008338 | PLCB1 | 13 | 789,380 | 1,695,139 | 3 | 5 |
| ENSBTAG00000000309 | PTPRT | 13 | 71,397,829 | 71,678,072 | 1 | 2 |
| ENSBTAG00000008279 | FRMD4A | 13 | 28,631,455 | 28,961,637 | 1 | 2 |
| ENSBTAG00000009475 | PLXDC2 | 13 | 21,595,581 | 22,015,323 | 1 | 1 |
| ENSBTAG00000027412 | SOD1 | 13 | 51,930,067 | 51,930,888 | 1 | 1 |
| ENSBTAG00000008969 | SLC9A8 | 13 | 78,628,908 | 78,698,768 | 1 | 1 |
| ENSBTAG00000011908 | CPQ | 14 | 69,287,302 | 69,893,052 | 2 | 2 |
| ENSBTAG00000004518 | GRHL2 | 14 | 64,905,620 | 65,031,370 | 1 | 1 |
| ENSBTAG00000015229 | DNAJC5B | 14 | 32,050,488 | 32,143,515 | 1 | 1 |
| ENSBTAG00000013033 | BTBD10 | 15 | 39,778,550 | 39,840,041 | 1 | 1 |
| ENSBTAG00000002382 | DDX10 | 15 | 18,644,692 | 18,952,411 | 1 | 1 |
| ENSBTAG00000003955 | MYO7A | 15 | 57,332,143 | 57,419,714 | 1 | 1 |
| ENSBTAG00000037384 | OR10AB1P | 15 | 45,478,430 | 45,479,365 | 1 | 1 |
| ENSBTAG00000000727 | RNF169 | 15 | 54,840,266 | 54,923,335 | 1 | 1 |
| ENSBTAG00000047461 | DYNC2H1 | 15 | 5,313,517 | 5,463,876 | 1 | 1 |
| ENSBTAG00000020046 | ASAM | 15 | 34,226,369 | 34,332,243 | 1 | 1 |
| ENSBTAG00000037661 | DENND1B | 16 | 78,480,806 | 78,590,234 | 1 | 2 |
| ENSBTAG00000002164 | AXDND1 | 16 | 62,138,403 | 62,214,479 | 1 | 2 |
| ENSBTAG00000033180 | SMYD3 | 16 | 31,589,396 | 32,333,109 | 1 | 2 |
| ENSBTAG00000023144 | PTPRC | 16 | 79,522,522 | 79,592,696 | 2 | 2 |
| ENSBTAG00000004407 | KCNK2 | 16 | 69,865,018 | 70,097,416 | 1 | 1 |
| ENSBTAG00000016542 | LAMB3 | 16 | 75,567,714 | 75,610,920 | 1 | 1 |
| ENSBTAG00000006188 | USH2A | 16 | 19,573,856 | 20,502,175 | 1 | 2 |
| ENSBTAG00000017561 | HHIPL2 | 16 | 26,713,225 | 26,741,364 | 1 | 1 |
| ENSBTAG00000024555 | EFCAB2 | 16 | 32,961,375 | 33,072,297 | 1 | 1 |
| ENSBTAG00000046256 | TMEM132C | 17 | 49,445,324 | 49,739,129 | 1 | 2 |
| ENSBTAG00000012738 | ZNF827 | 17 | 12,587,397 | 12,739,481 | 1 | 1 |
| ENSBTAG00000002531 | ARHGAP10 | 17 | 10,182,664 | 10,560,361 | 1 | 1 |
| ENSBTAG00000002813 | GAB1 | 17 | 14,625,987 | 14,753,591 | 1 | 1 |
| ENSBTAG00000003447 | ZNF551 | 18 | 65,291,985 | 65,319,947 | 1 | 2 |
| ENSBTAG00000002287 | CHD9 | 18 | 21,726,409 | 21,857,238 | 1 | 1 |
| ENSBTAG00000003334 | ADAP2 | 19 | 18,353,810 | 18,388,312 | 1 | 1 |
| ENSBTAG00000044618 | SNORA31 | 19 | 3,899,300 | 3,899,416 | 1 | 1 |
| ENSBTAG00000022509 | DNAH9 | 19 | 30,963,518 | 31,248,936 | 1 | 1 |
| ENSBTAG00000027074 | SV2B | 21 | 16,214,670 | 16,460,490 | 2 | 3 |
| ENSBTAG00000047543 | NTRK3 | 21 | 19,493,058 | 19,520,421 | 1 | 1 |
| ENSBTAG00000007382 | SCAPER | 21 | 32,118,828 | 32,512,606 | 1 | 1 |
| ENSBTAG00000010416 | RIN3 | 21 | 57,859,148 | 57,953,844 | 1 | 1 |
| ENSBTAG00000013047 | GRM7 | 22 | 18,740,484 | 19,647,747 | 1 | 6 |
| ENSBTAG00000012073 | VOPP1 | 22 | 495,506 | 677,104 | 1 | 1 |
| ENSBTAG00000003592 | CNTN6 | 22 | 25,053,636 | 25,233,677 | 1 | 1 |
| ENSBTAG00000034496 | SHFM1 | 22 | 22,098,224 | 22,098,436 | 1 | 1 |
| ENSBTAG00000007360 | TMEM43 | 22 | 58,726,302 | 58,747,763 | 1 | 1 |
| ENSBTAG00000003359 | ELOVL5 | 23 | 25,155,743 | 25,228,997 | 1 | 1 |
| ENSBTAG00000013831 | DSG1 | 24 | 26,089,489 | 26,133,405 | 1 | 1 |
| ENSBTAG00000009822 | PPP4R1 | 24 | 42,093,001 | 42,121,847 | 1 | 1 |
| ENSBTAG00000000390 | TPST1 | 25 | 28,339,035 | 28,445,471 | 1 | 1 |
| ENSBTAG00000016244 | VWA3A | 25 | 19,992,453 | 20,049,454 | 1 | 1 |
| ENSBTAG00000004077 | YWHAG | 25 | 34,884,283 | 34,906,639 | 1 | 1 |
| ENSBTAG00000045905 | PCDH15 | 26 | 5,017,714 | 5,578,654 | 1 | 8 |
| ENSBTAG00000007948 | SORCS1 | 26 | 27,810,198 | 28,389,391 | 3 | 6 |
| ENSBTAG00000022715 | DMBT1 | 26 | 42,782,329 | 42,813,472 | 1 | 1 |
| ENSBTAG00000037795 | CYP2C87 | 26 | 16,030,292 | 16,065,395 | 1 | 1 |
| ENSBTAG00000004830 | ADAM18 | 27 | 34,517,618 | 34,625,508 | 1 | 3 |
| ENSBTAG00000033137 | PSD3 | 27 | 38,483,892 | 38,797,972 | 1 | 2 |
| ENSBTAG00000020361 | SLC35F3 | 28 | 6,762,322 | 7,195,661 | 1 | 5 |
| ENSBTAG00000011072 | ADK | 28 | 30,215,525 | 30,732,444 | 1 | 2 |
| ENSBTAG00000021497 | CDH23 | 28 | 27,729,164 | 28,123,449 | 1 | 1 |
| ENSBTAG00000004081 | FAT3 | 29 | 1,965,869 | 2,605,125 | 1 | 4 |
| ENSBTAG00000001043 | MGC157332 | 29 | 20,259,769 | 20,557,376 | 1 | 1 |
| ENSBTAG00000016506 | ST3GAL-IV | 29 | 30,073,042 | 30,112,563 | 1 | 1 |
