# Supplementary material for: Deleted copy number variation of Hanwoo and Holstein using next generation sequencing at the population level
Source: BMC Genomics. 2014 Mar 27;15:240. doi: 10.1186/1471-2164-15-240 (PMC4051123; doi:10.1186/1471-2164-15-240)
Supplement: Additional file 11 — Genes that overlapped with Holstein breed-specific CNVs. [file 1471-2164-15-240-S11.DOCX]

**Additional File 11. Genes that overlapped with Holstein breed-specific CNVs**

| Ensemble Gene | Gene Symbol | Chr | Gene Start | Gene End | # Breed  Specific CNV | # CNV |
| --- | --- | --- | --- | --- | --- | --- |
| ENSBTAG00000003936 | PNKD | 2 | 107,020,657 | 107,093,550 | 1 | 1 |
| ENSBTAG00000017056 | PKLR | 3 | 15,399,755 | 15,408,994 | 1 | 1 |
| ENSBTAG00000017055 | HCN3 | 3 | 15,409,813 | 15,418,775 | 1 | 1 |
| ENSBTAG00000019027 | SLC30A7 | 3 | 42,465,567 | 42,559,234 | 1 | 1 |
| ENSBTAG00000004023 | KIAA1324L | 4 | 33,518,953 | 33,763,007 | 1 | 2 |
| ENSBTAG00000020247 | ADCYAP1R1 | 4 | 65,670,543 | 65,729,276 | 1 | 1 |
| ENSBTAG00000046430 | ZNF804B | 4 | 73,326,980 | 73,897,041 | 1 | 1 |
| ENSBTAG00000032183 | NELL2 | 5 | 35,657,329 | 36,042,694 | 1 | 2 |
| ENSBTAG00000020679 | CNTN1 | 5 | 39,998,363 | 40,264,645 | 1 | 1 |
| ENSBTAG00000010149 | CRY1 | 5 | 70,606,115 | 70,701,030 | 1 | 1 |
| ENSBTAG00000006434 | SYNPO2 | 6 | 7,388,728 | 7,590,933 | 1 | 3 |
| ENSBTAG00000016515 | EFNA5 | 7 | 109,049,590 | 109,217,439 | 1 | 4 |
| ENSBTAG00000015335 | BAI3 | 9 | 7,914,952 | 8,455,993 | 1 | 2 |
| ENSBTAG00000007758 | PDE10A | 9 | 101,987,619 | 102,068,801 | 1 | 2 |
| ENSBTAG00000005016 | AP3B1 | 10 | 9,040,253 | 9,300,567 | 1 | 1 |
| ENSBTAG00000005751 | CDAN1 | 10 | 38,138,863 | 38,151,656 | 1 | 1 |
| ENSBTAG00000021102 | GALM | 11 | 21,040,425 | 21,095,033 | 1 | 1 |
| ENSBTAG00000020893 | MATN3 | 11 | 78,889,151 | 78,907,349 | 1 | 1 |
| ENSBTAG00000002137 | SUGT1 | 12 | 11,131,393 | 11,172,149 | 1 | 1 |
| ENSBTAG00000013114 | ZMYND8 | 13 | 76,501,073 | 76,625,012 | 1 | 1 |
| ENSBTAG00000008571 | CUX2 | 17 | 57,065,816 | 57,343,548 | 1 | 1 |
| ENSBTAG00000023541 | C6ORF10 | 23 | 26,726,417 | 26,794,259 | 1 | 2 |
| ENSBTAG00000004940 | BRUNOL4 | 24 | 19,819,065 | 20,129,055 | 1 | 1 |
| ENSBTAG00000021856 | C10ORF28 | 26 | 19,164,410 | 19,197,680 | 1 | 1 |
| ENSBTAG00000033137 | PSD3 | 27 | 38,483,892 | 38,797,972 | 1 | 2 |
| ENSBTAG00000020361 | SLC35F3 | 28 | 6,762,322 | 7,195,661 | 1 | 5 |
