# Supplementary material for: Deleted copy number variation of Hanwoo and Holstein using next generation sequencing at the population level
Source: BMC Genomics. 2014 Mar 27;15:240. doi: 10.1186/1471-2164-15-240 (PMC4051123; doi:10.1186/1471-2164-15-240)
Supplement: Additional file 12 — Gene description and references for genes related to phosphorylation or protein modification process in Hanwoo. Gene description and references of some of the genes related to phosphorylation or protein modification process from GO analysis results of genes that overlapped with Hanwoo breed-specific CNV. These genes were identified in both this study and previous studies. [file 1471-2164-15-240-S12.DOCX]

**Additional File 12. Gene description and references for genes related to phosphorylation or protein modification process in Hanwoo**

| Gene | Chr | Reference | Description |
| --- | --- | --- | --- |
| NDUFA10 | chr3 | Perez, Cañón et al. 2010 | *NDUFA10* is associated with long-chain omega-3 fatty acids in bovine skeletal muscle. |
| WNK1 | chr5 | Moore, Garg et al. 2000 | WNK1 encodes Serine/threonine-protein kinase which plays an important role in cell proliferation and in actin cytoskeletal reorganization. |
| MAPK10 | chr6 | Fluckey, Knox et al. 2006 | MAPK10 are related to the Mitogen-Activated Protein Kinase (MAPK) system, which is a major growth signaling pathway that controls skeletal muscle growth. |
| FER | chr7 | Aspenström 1997 | FER encodes FER tyrosine kinase, which may acts downstream of cell surface receptors for growth factors and plays a role in the regulation of the actin cytoskeleton. |
| RPS6KA2 | chr9 | Yin, Kim et al. 2007 | RPS6KA2 encodes a member of the RSK (ribosomal S6 kinase) family of serine/threonine kinases which has been implicated in controlling cell growth and differentiation. |
| MAP4K3 | chr11 | Fluckey, Knox et al. 2006 | MAP4K3 are related to the Mitogen-Activated Protein Kinase (MAPK) system, which is a major growth signaling pathway that controls skeletal muscle growth. |
| PTPRT | chr13 | Sun and Tonks 1994 | The proteins encoded by PTPRT are members of the protein tyrosine phosphatase (PTP) family that are known to be signaling molecules that regulate cell growth. |
| PTPRC | chr16 | Sun and Tonks 1994 | The proteins encoded by PTPRC are members of the protein tyrosine phosphatase (PTP) family that are known to be signaling molecules that regulate cell growth. |
| GAB1 | chr17 | Rodrigues, Falasca et al. 2000 | GAB1 encodes a bound protein 2-associated protein that plays a central role in cellular growth. |
| NTRK3 | chr21 | Lannon and Sorensen 2005 | NTRK3 encodes a member of the neurotrophic tyrosine receptor kinase (NTRK) family which plays a role in cell growth, development, and cell survival. |
