# Supplementary material for: Deleted copy number variation of Hanwoo and Holstein using next generation sequencing at the population level
Source: BMC Genomics. 2014 Mar 27;15:240. doi: 10.1186/1471-2164-15-240 (PMC4051123; doi:10.1186/1471-2164-15-240)
Supplement: Additional file 13 — Gene description and references for genes related to cell adhesion and maintenance in Hanwoo. Gene description and references of some of the genes related to cell adhesion and maintenance from GO analysis results of genes that overlapped with Hanwoo-specific CNV. These genes were identified in this study and previous studies. [file 1471-2164-15-240-S13.DOCX]

**Additional File 13. Gene description and references for genes related to cell adhesion and maintenance in Hanwoo**

| Gene | Chr | Reference | Gene Description |
| --- | --- | --- | --- |
| ITGAV | chr2 | Takada, Ye et al. 2007 | ITGAV encodes a protein that is a member of the integrin superfamily which interacts with several extracellular matrix proteins to mediate cell adhesion. |
| COL28A1 | chr4 | Veit, Kobbe et al. 2006 | COL28A1 belongs to a class of collagens containing von Willebrand factor. |
| FER | chr7 | Rosato, Veltmaat et al. 1998 | Fer protein regulates cell-cell adhesion. |
| TLN2 | chr10 | Senetar, Moncman et al. 2007 | This gene encodes a protein related to talin 1, a cytoskeletal protein that plays a significant role in the assembly of  actin filaments and may play an important role in cell adhesion. |
| LAMB3 | chr16 | Vidal, Baudoin et al. 1995 | In biological process related to collagen, LAMB3 product mediates the attachment, migration and organization of cells into tissues by interacting with other extracellular matrix components. |
| DSG1 | chr24 | Runswick, O'Hare et al. 2001 | DSG1 encodes desmosomal glycoprotein and desmosomal adhesion as these genes regulate intercellular junctions of epithelia. |
| PCDH15 | chr26 | Halbleib and Nelson 2006 | PCDH15 encodes protocadherin 15, a member of the cadherin superfamily which related to cell adhesion. |
| CDH23 | chr28 | Marthiens, Gavard et al. 2002 | CDH23 encode Cadherin-23, a calcium dependent cell-cell adhesion glycoprotein and previous study implicated this cadherin in myogenesis. |
| FAT3 | chr29 | Mitsui, Nakajima et al. 2002 | Fat3 protein l may be involved in cell adhesion |
