# Supplementary material for: Deleted copy number variation of Hanwoo and Holstein using next generation sequencing at the population level
Source: BMC Genomics. 2014 Mar 27;15:240. doi: 10.1186/1471-2164-15-240 (PMC4051123; doi:10.1186/1471-2164-15-240)
Supplement: Additional file 14 — Gene description and references for genes related to dairy production in Holstein. Gene description and references of some of the genes related to dairy production. These genes overlapped with Hanwoo breed-specific CNV and were identified in this study and previous studies. [file 1471-2164-15-240-S14.DOCX]

**Additional File 14. Gene description and references for genes related to dairy production in Holstein**

| Gene | Chr | Reference | Gene Description |
| --- | --- | --- | --- |
| PKLR | chr3 | Baik, Etchebarne et al. 2009 | PKLR encoding pyruvate kinase was found to have expression in mammary tissue of lactating dairy cow. |
| ADCYAP1R1 | chr4 | Winters and Moore 2011 | ADCYAP1R1 encodes type I adenylate cyclase activating polypeptide receptor, which may regulate the release of prolactin. |
| NELL2 | chr5 | Connor, Siferd et al. 2008 | NELL2 encodes Neural Epidermal Growth Factor-Like 2, which is down regulated in the bovine mammary gland and affects milking frequency. |
| CRY1 | chr5 | Casey and Plaut 2012 | CRY1 encodes a flavin adenine dinucleotide-binding protein that is a key component of the circadian core that affected development of the mammary gland and lactation. |
| EFNA5 | chr7 | Li, Wang et al. 2010 | EFNA5 is a member of the ephrin gene family and was previously reported to be the top milk production trait SNP in Canadian Holstein cattle. |
| PDE10A | chr9 | Dostaler-Touchette et al. 2009 | The protein encoded by PDE10A belongs to the cyclic nucleotide phosphodiesterase family and PDE10 appear to be functional in the bovine mammary gland. |
| AP3B1 | chr10 | Bionaz, Periasamy et al. 2012 | The protein encoded by AP3B1 interacts with the scaffolding protein clathrin, which is important for lactating mammary glands as lactation secretes milk components through vesicles in dairy cattle. |
| GALM | chr11 | D'Alessandro, Zolla et al. 2011 | The protein encoded by GALM is expressed in the cytoplasm and has a preference for galactose and contained in networks of bovine milk proteins. |
| MATN3 | chr11 | Yucesoy, Charles et al. 2013 | MATN3 is related to genetic risk factors for osteoarthritis which related to dairy production. |
| C6ORF10 | chr23 | Sadkowski, Jank et al. 2009 | Transcriptional profiles of dairy and beef breeds bulls showed that C10ORF28 is down-regulated in Holstein |
