# Supplementary material for: Deleted copy number variation of Hanwoo and Holstein using next generation sequencing at the population level
Source: BMC Genomics. 2014 Mar 27;15:240. doi: 10.1186/1471-2164-15-240 (PMC4051123; doi:10.1186/1471-2164-15-240)

BovineCNV0531, 319 bp – 1,362 bp

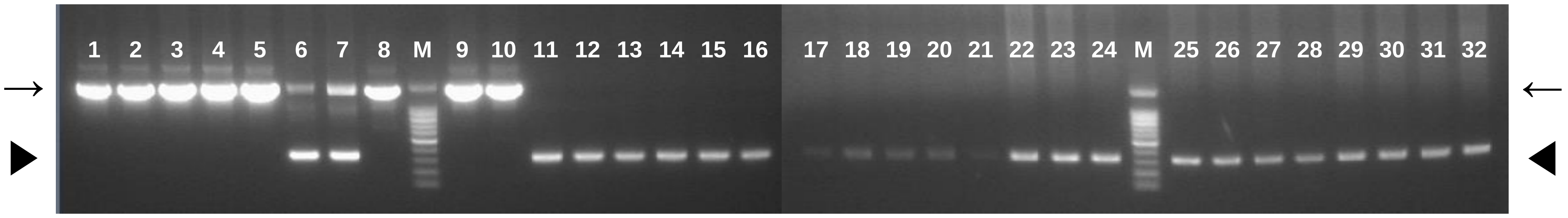

BovineCNV5282, 507 bp – 1,179 bp

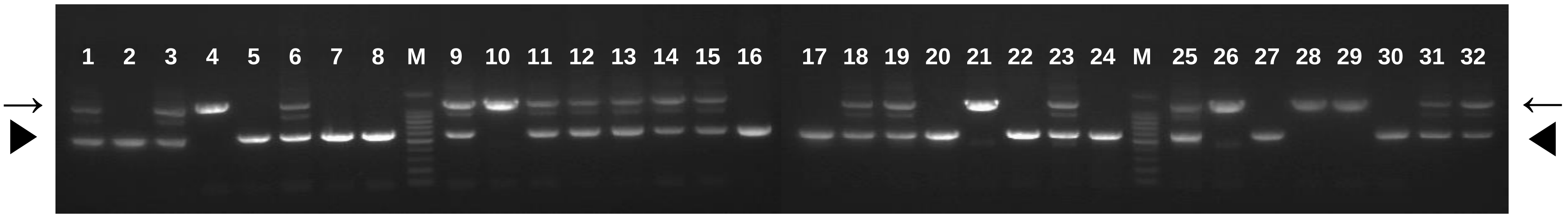

BovineCNV5283, 214 bp – 463 bp

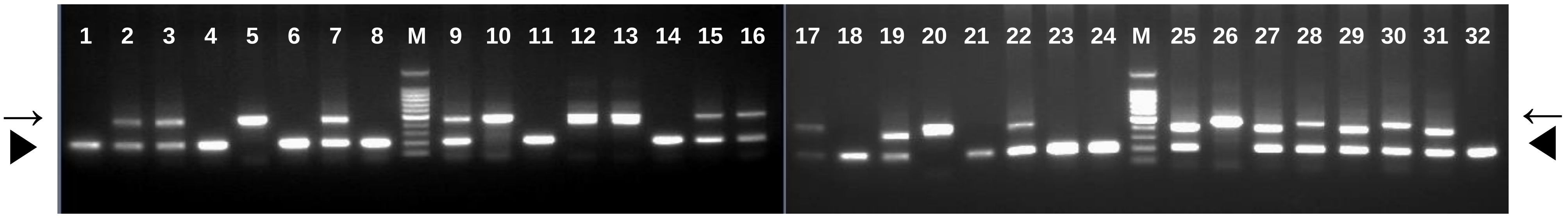

BovineCNV3795, 360 bp – 1,466 bp

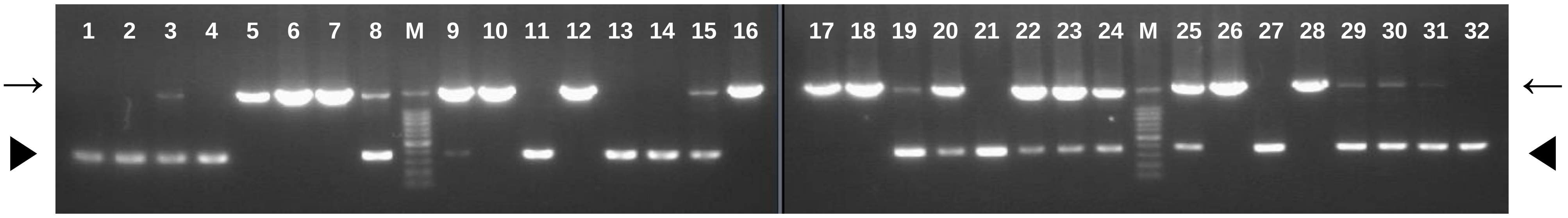

BovineCNV3797, 609 bp – 821 bp

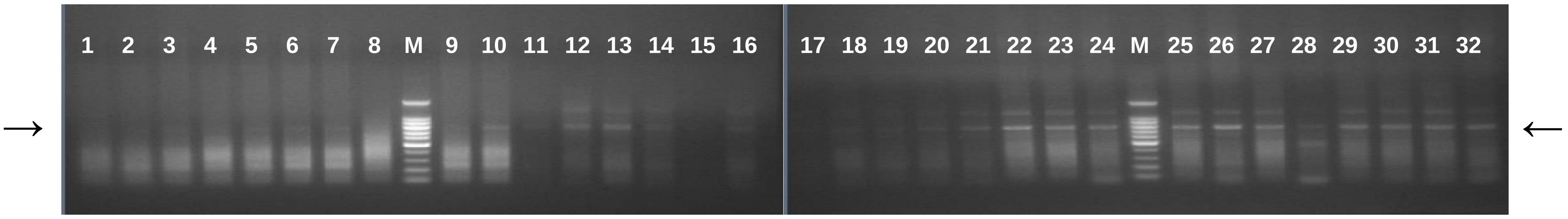

BovineCNV0050, 342 bp – 1,668 bp

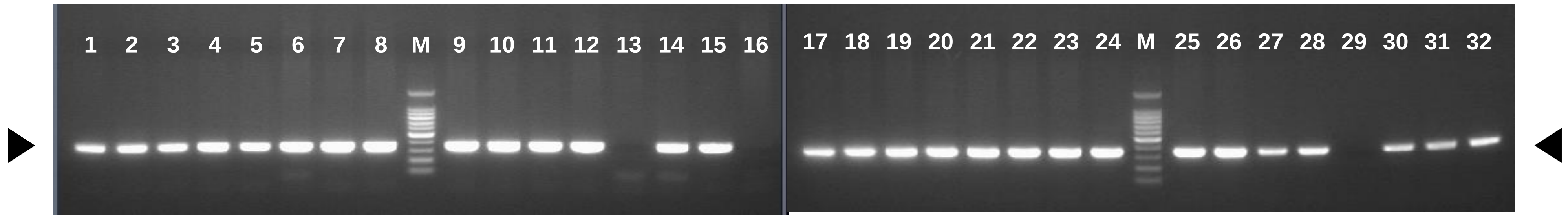

BovineCNV0051, 287 bp – 891 bp

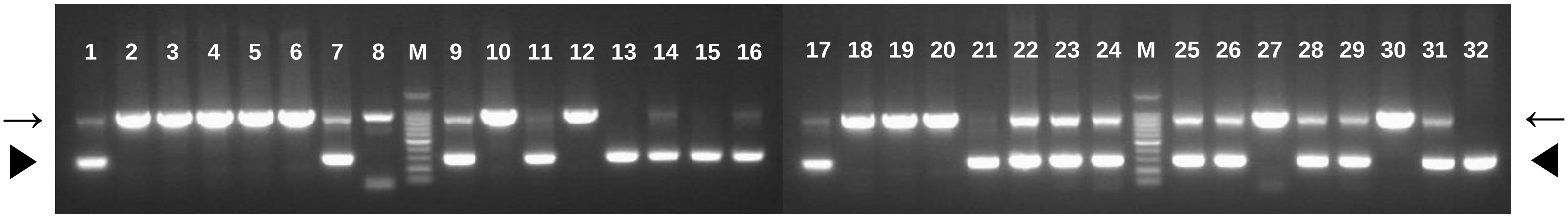

BovineCNV3226, 582 bp – 1,282 bp

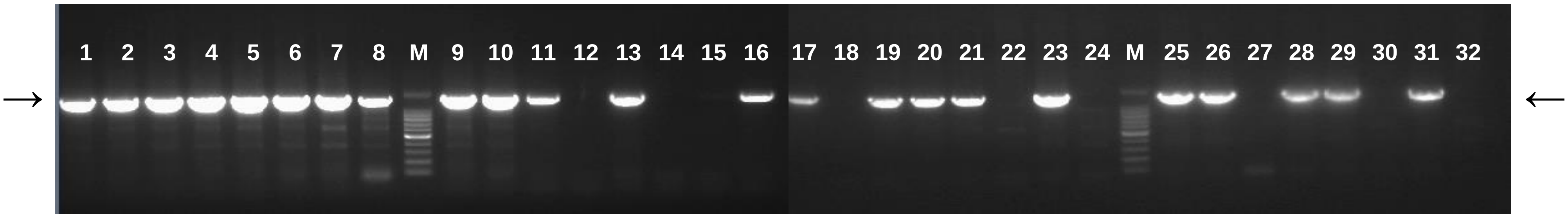

BovineCNV3227, 357 bp – 591 bp

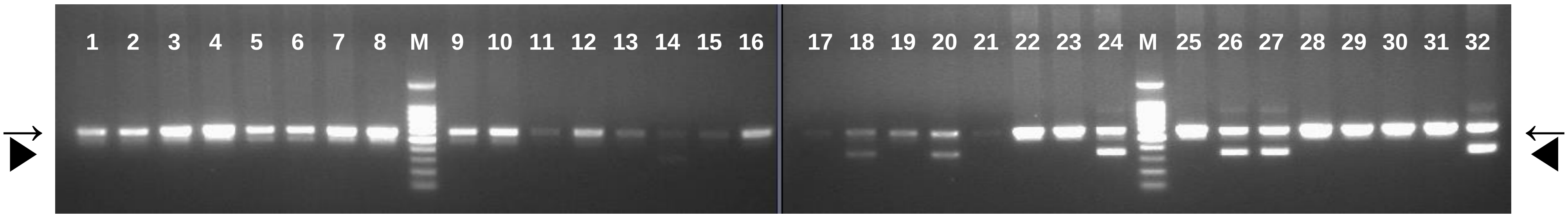

BovineCNV3228, 374 bp – 1,678 bp

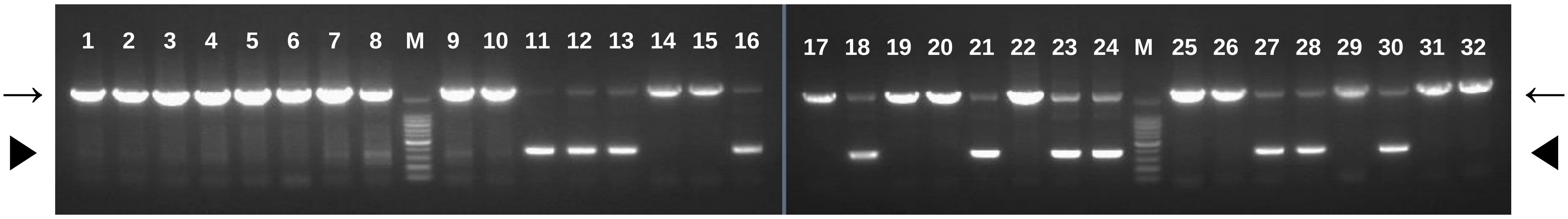

BovineCNV3229, 482 bp – 869 bp

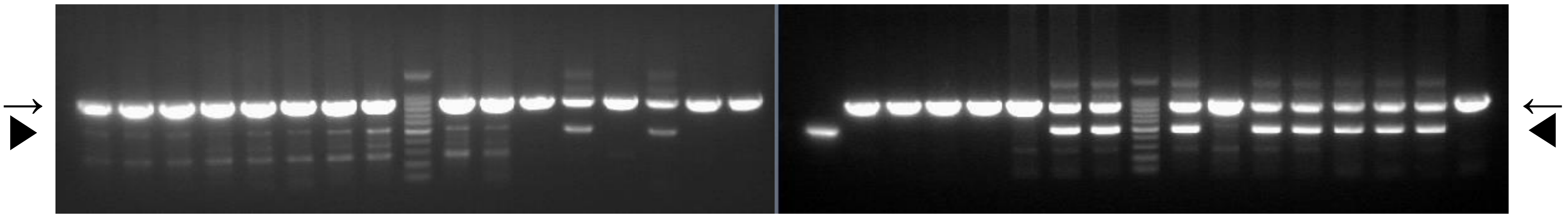

BovineCNV3230, 302 bp – 1,523 bp

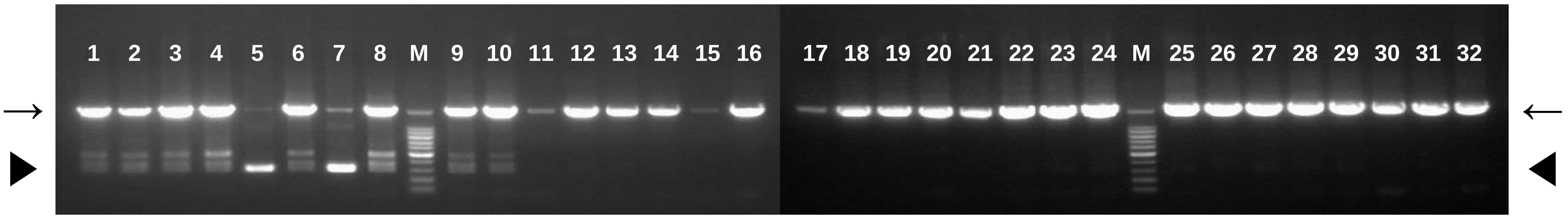

BovineCNV2505, 440 bp – 669 bp

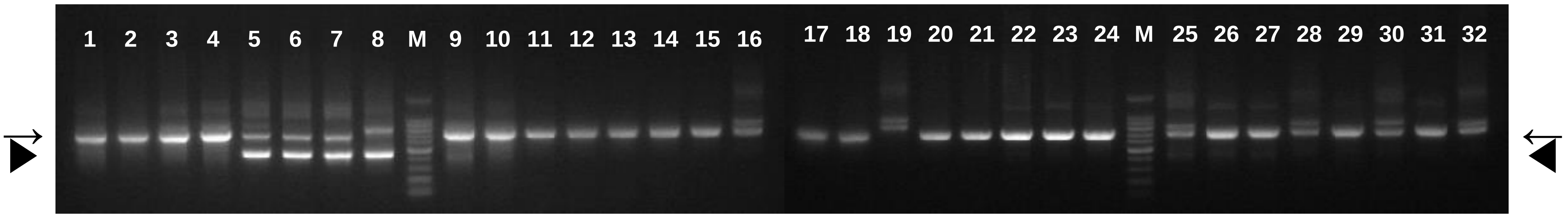

BovineCNV2506, 369 bp – 658 bp

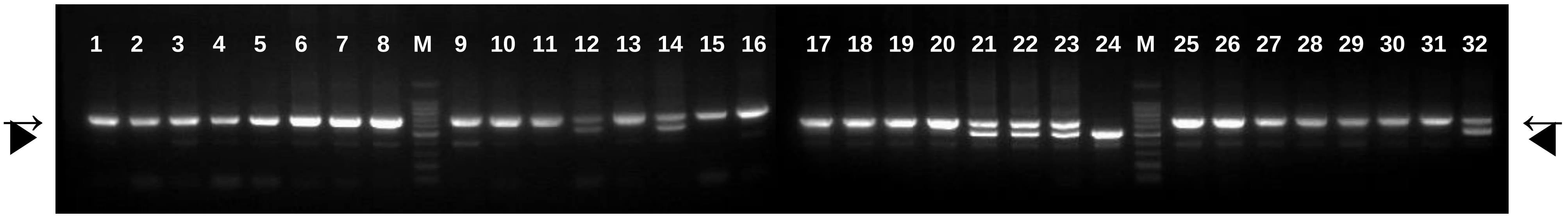

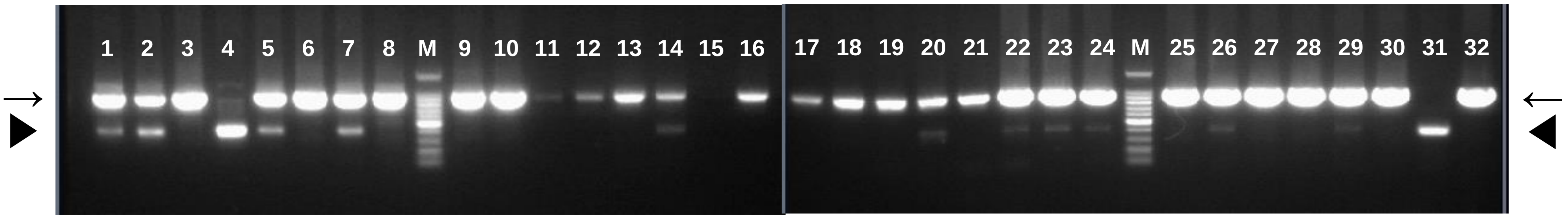

BovineCNV6287, 434 bp – 1,395 bp

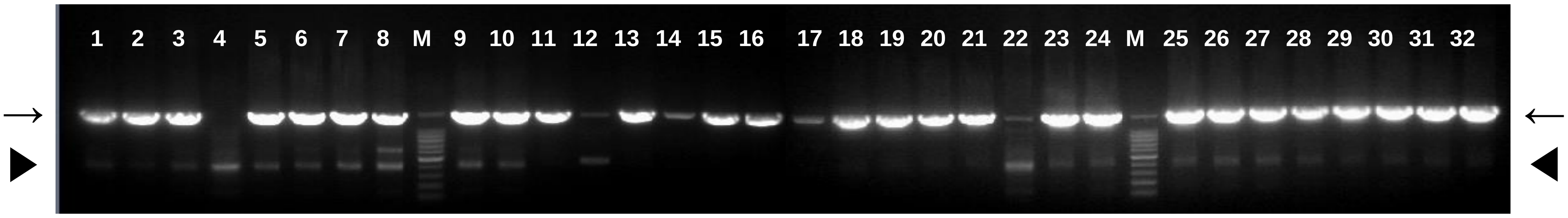

BovineCNV6288, 718 bp – 2,008 bp

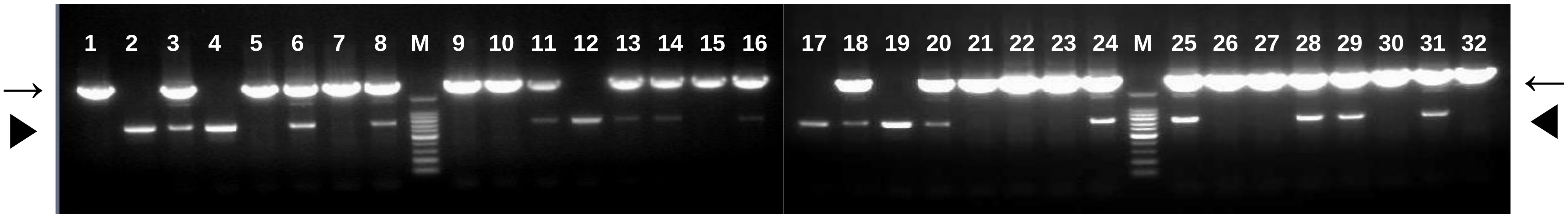

BovineCNV6289, 399 bp – 971 bp

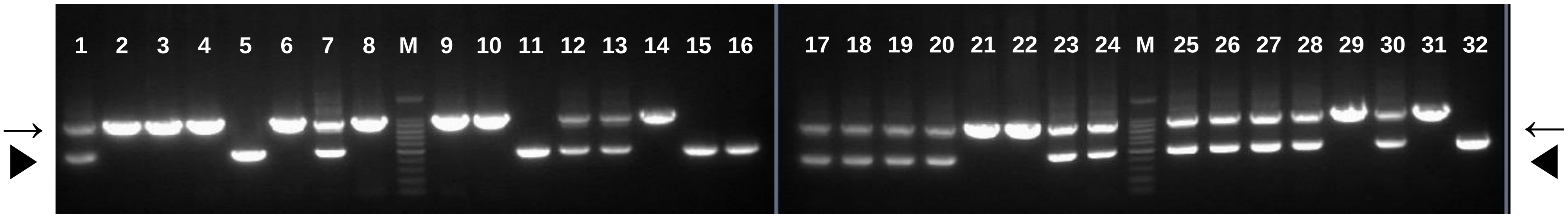

BovineCNV6290, 478 bp – 969 bp

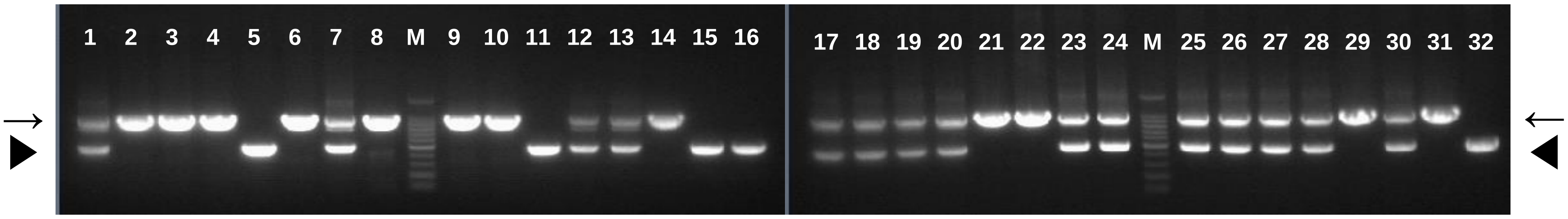

Supplement: Additional file 15 — Genomic DNA amplification in 19 CNV regions. Genomic DNA from 32 individuals (1–10, Holstein; 11–32, Hanwoo) was used for PCR amplification for validating 19 CNV regions. M indicates a 100 bp-DNA ladder. Arrow and arrowheads show deleted allele and non-deleted allele amplicon, respectively. Predicted length of the PCR products from the deleted and non-deleted alleles are shown in the top left panel of each gel image. [file 1471-2164-15-240-S15.PDF]
