# Supplementary material for: Deleted copy number variation of Hanwoo and Holstein using next generation sequencing at the population level
Source: BMC Genomics. 2014 Mar 27;15:240. doi: 10.1186/1471-2164-15-240 (PMC4051123; doi:10.1186/1471-2164-15-240)
Supplement: Additional file 16 — Genotype comparison between result from genomic DNA amplification and GenomeSTRiP. The genotype of each CNV and individual are summarized by a heat map. The genotype of the examined 19 CNV regions by PCR was compared to that of the GenomeSTRiP result. Matching score was calculated by dividing the number of individual, whose predicted genotype was the same as the PCR result, to the total number of individuals examined (n = 32). As the CNVs detecting only deleted or non-deleted alleles (BovineCNV0050, BovineCNV3226, and BovineCNV3797) showed limited accuracy, a lower score of 0.7 was used as the matching score. [file 1471-2164-15-240-S16.PDF]

CNV Number	Holstein										Hanwoo																							Matching rate (%)
	1	2	3	4	5	6	7	8	9	10	11	12	13	14	15	16	17	18	19	20	21	22	23	24	25	26	27	28	29	30	31	32		
BovineCNV0531																																	Predicted	100
																																	PCR	
BovineCNV5282																																	Predicted	93.75
																																	PCR	
BovineCNV5283																																	Predicted	96.875
																																	PCR	
BovineCNV3795																																	Predicted	96.875
																																	PCR	
BovineCNV3797																																	Predicted	37.1875
	X	X	X	X				X	X																								PCR	
BovineCNV0050																																	Predicted	63.4375
												X				X													X				PCR	
BovineCNV0051																																	Predicted	78.125
																																	PCR	
BovineCNV3226																																	Predicted	70
												X		X				X				X		X			X			X		X	PCR	
BovineCNV3227																																	Predicted	53.125
																																	PCR	
BovineCNV3228																																	Predicted	75
																																	PCR	
BovineCNV3229																																	Predicted	78.125
																																	PCR	
BovineCNV3230																																	Predicted	68.75
																																	PCR	
BovineCNV2505																																	Predicted	93.75
																		X															PCR	
BovineCNV2506																																	Predicted	68.75
																																	PCR	
BovineCNV6286																																	Predicted	78.125
															X																		PCR	
BovineCNV6287																																	Predicted	93.75
				X																													PCR	
BovineCNV6288																																	Predicted	100
																																	PCR	
BovineCNV6289																																	Predicted	87.5
																																	PCR	
BovineCNV6290																																	Predicted	87.5
																																	PCR	
