# Supplementary figures and images for: Deleted copy number variation of Hanwoo and Holstein using next generation sequencing at the population level
Source: BMC Genomics. 2014 Mar 27;15:240. doi: 10.1186/1471-2164-15-240 (PMC4051123; doi:10.1186/1471-2164-15-240)

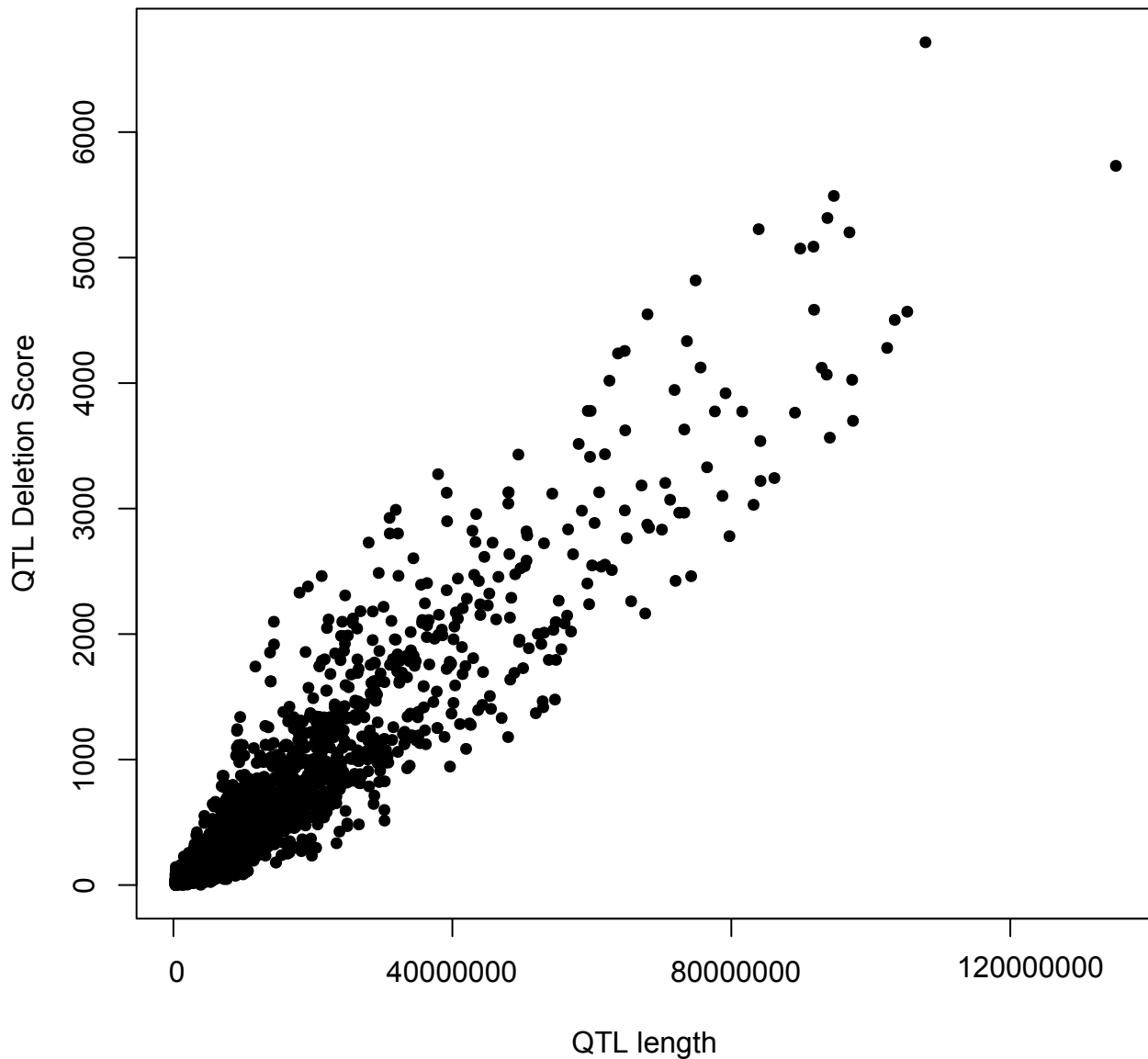

Supplement: Additional file 17 — Relationships between QTL length and QTL deletion score. [file 1471-2164-15-240-S17.PDF]

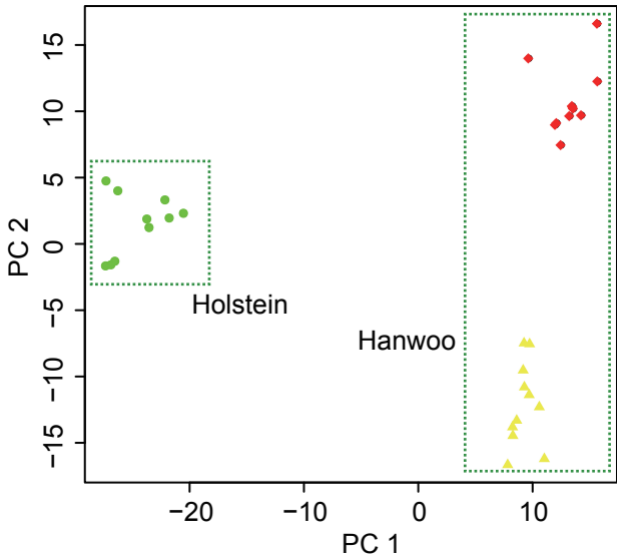

Supplement: Additional file 18 — PCA using all deleted cattle CNV as markers. Green circle represents Holstein and other two colors represent the two different Hanwoo populations. Red diamond represents Hanwoo from RDA in Suwon and yellow represents Hanwoo from Kyungpook National University. [file 1471-2164-15-240-S18.PDF]

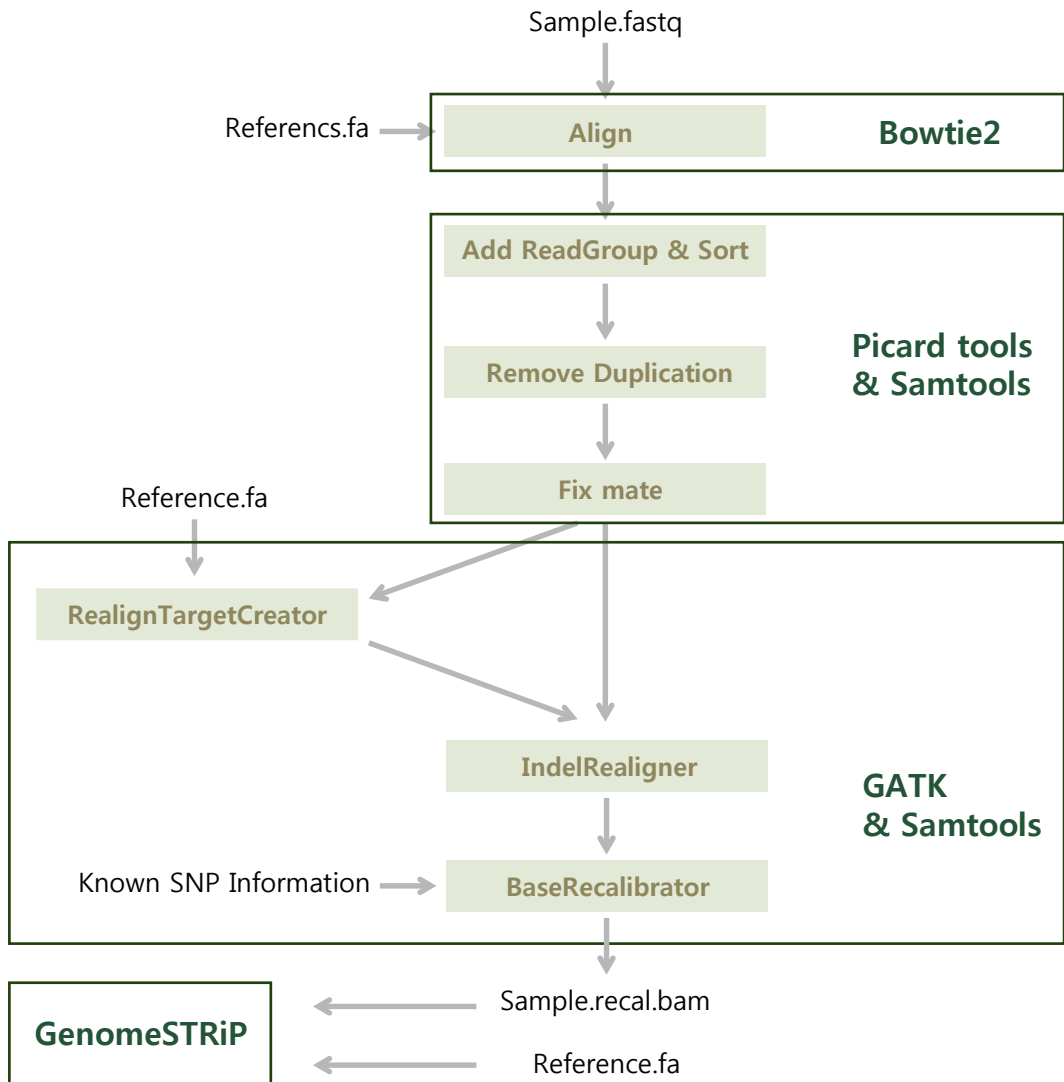

Supplement: Additional file 20 — Resequencing NGS data process pipeline before Genome STRiP for CNV extraction. [file 1471-2164-15-240-S20.PDF]

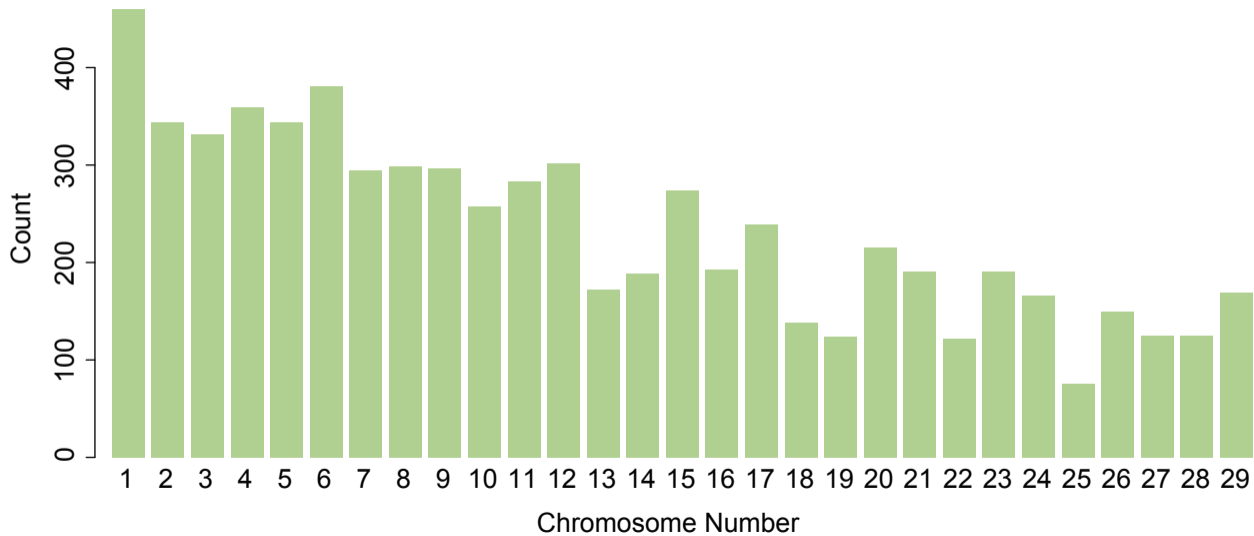

Supplement: Additional file 21 — Distribution per chromosome of the deleted CNV on the cattle genome. [file 1471-2164-15-240-S21.PDF]

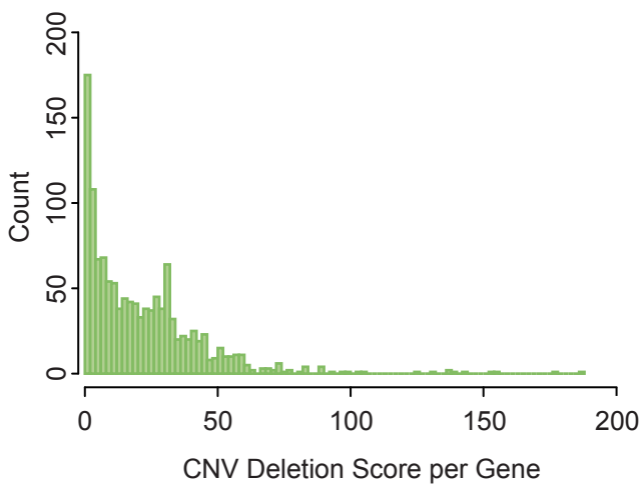

Supplement: Additional file 22 — Distribution of the deletion score for the bovine genes. [file 1471-2164-15-240-S22.PDF]

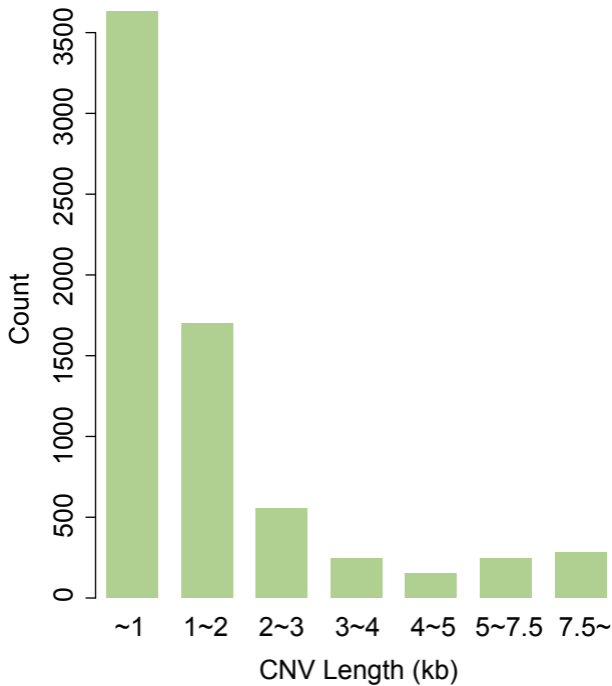

Supplement: Additional file 23 — Histogram of the bovine deleted CNV length. [file 1471-2164-15-240-S23.PDF]

0.2

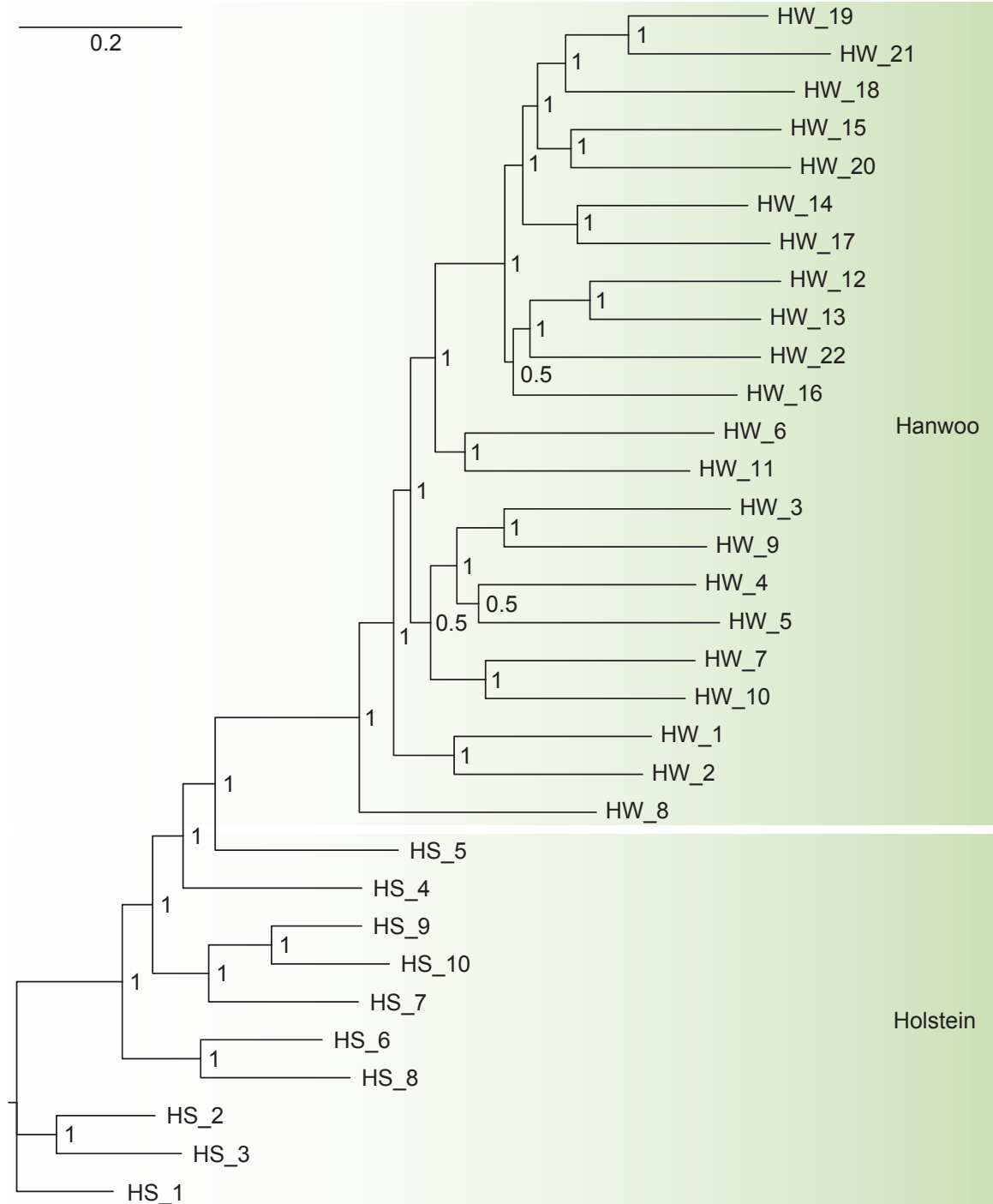

Supplement: Additional file 24 — Phylogenetic analysis using Bayesian Inference. Sample ID for each branch is in Additional file 1. [file 1471-2164-15-240-S24.PDF]
