# Supplementary material for: Deleted copy number variation of Hanwoo and Holstein using next generation sequencing at the population level
Source: BMC Genomics. 2014 Mar 27;15:240. doi: 10.1186/1471-2164-15-240 (PMC4051123; doi:10.1186/1471-2164-15-240)
Supplement: Additional file 25 — Information of the primer pairs used for zygosity validation. [file 1471-2164-15-240-S25.DOCX]

**Additional File 18. Information of the primer pairs used for zygosity validation**

| Gene Symbol | CNV Number | Primer (5´ to 3´)^*^ | | Predicted CNV length (bp)** | Amplicon Size (bp) | | PCR condition | |
| --- | --- | --- | --- | --- | --- | --- | --- | --- |
|  |  |  |  |  | CNV  deletion | CNV  Non-deletion | Anealing  Temperature | Extention  time in 72 °C |
| *TTN* | BovineCNV0531 | F | TGGGGGAAACCATCATAAAC | 1043 | 319 | 1362 | 60 | 1 min |
|  |  | R | CCACACAGGATTTGAACCATC |  |  |  |  |  |
| *SLIT3* | BovineCNV5282 | F | TGAGGGACAGAGACAGAGCA | 609 | 570 | 1179 | 66 | 1 min |
|  |  | R | TCCAGTTGAGCTGAGTTGAGG |  |  |  |  |  |
|  | BovineCNV5283 | F | GACATTCGCTTGGTTGGCTG | 249 | 214 | 463 | 60 | 1 min |
|  |  | R | GCTTTGAGATTGCTTCATTTCCC |  |  |  |  |  |
| *KLHL1* | BovineCNV3795 | F | TCATGCTTAGAACTTTCCCCGTT | 1106 | 360 | 1466 | 64 | 1 min |
|  |  | R | CGCTTGCTTCAGCTTAGCCTT |  |  |  |  |  |
|  | BovineCNV3797 | F | CAATCAATGGGGTCACAAAG | 212 | 609 | 821 | 64 | 1 min |
|  |  | R | AAAGGCTGGGAAAGGAAGAG |  |  |  |  |  |
| *NCAM2* | BovineCNV0050 | F | ATTCATGGAGAAAAATGCTTGCC | 1326 | 342 | 1668 | 65 | 1min 30sec |
|  |  | R | GCTGGTTGGTCATAGCCTGAGTT |  |  |  |  |  |
|  | BovineCNV0050_In^†^ | F | ATTCATGGAGAAAAATGCTTGCC | 1326 | N.D | 1019 | 64 | 1 min |
|  |  | R | GCTGGTTGGTCATAGCCTGAGTT |  |  |  |  |  |
|  | BovineCNV0051 | F | GCCTCCAGCAAACTTACAGACAT | 604 | 287 | 891 | 60 | 1 min |
|  |  | R | TTTTCACAAAGAGAACCAGAAGCA |  |  |  |  |  |
| *MDGA2* | BovineCNV3226 | F | CCCATCCTCAGAAATCCTTTA | 700 | 582 | 1282 | 58 | 1 min |
|  |  | R | GGTAAATGGGATTGATTCCTTG |  |  |  |  |  |
|  | BovineCNV3227 | F | CTACCATCTGGCCCTTCAAC | 234 | 357 | 591 | 64 | 1 min |
|  |  | R | CAAACATGGAAAGGAATCCAA |  |  |  |  |  |
|  | BovineCNV3228 | F | ATGATGTCTTCTGGGCAAGT | 1304 | 374 | 1678 | 58 | 1 min |
|  |  | R | TTTCGTCTGAGTGCTCCATC |  |  |  |  |  |
|  | BovineCNV3229 | F | TTAGTGCCCCTCTCCTTTCC | 385 | 482 | 867 | 60 | 1 min |
|  |  | R | GCCTTCCTTTCCAACATCAC |  |  |  |  |  |
|  | BovineCNV3230 | F | CCCCAGGCTCTTCTGTTCT | 1221 | 302 | 1523 | 64 | 1 min |
|  |  | R | TGTCAGTTTGTGATGAAAGTTGG |  |  |  |  |  |
| *EFNA5* | BovineCNV2505 | F | GGAACACAGACAACAGGCAGA | 229 | 440 | 669 | 64 | 1 min |
|  |  | R | AGGGGAAAGAAGGAGTGGAA |  |  |  |  |  |
|  | BovineCNV2506 | F | AAGAGATTCGGGAAGGGACT | 289 | 369 | 658 | 64 | 1 min |
|  |  | R | AAGAACGACACCTTGCTGCT |  |  |  |  |  |
| *PRKG1* | BovineCNV6286 | F | TCTCTTTCCCCCAATCTCAA | 475 | 404 | 879 | 60 | 1 min |
|  |  | R | CACAACATCACCACATCAAGG |  |  |  |  |  |
|  | BovineCNV6287 | F | GCAGCAAAAGAAGGGAAAGA | 961 | 434 | 1395 | 64 | 1 min |
|  |  | R | TGAAGCAACTGAAACCCAGA |  |  |  |  |  |
|  | BovineCNV6288 | F | GACACACAAAGGGAAATAGAGGA | 1290 | 718 | 2008 | 64 | 1min 30sec |
|  |  | R | GACAGTCTGATTGGCTGTTTG |  |  |  |  |  |
|  | BovineCNV6289 | F | ATGCTATGGAAACCGAGAGG | 572 | 399 | 971 | 60 | 1 min |
|  |  | R | AACTATGATGCCCAACTTCACA |  |  |  |  |  |
|  | BovineCNV6290 | F | ATGCTATGGAAACCGAGAGG | 491 | 478 | 969 | 60 | 1 min |
|  |  | R | CTATGATGCCCAACTTCACA |  |  |  |  |  |
