# Supplementary material for: Deleted copy number variation of Hanwoo and Holstein using next generation sequencing at the population level
Source: BMC Genomics. 2014 Mar 27;15:240. doi: 10.1186/1471-2164-15-240 (PMC4051123; doi:10.1186/1471-2164-15-240)
Supplement: Additional file 19 — CNV validation scheme by genomic DNA PCR. To validate the CNV extracted by GenomeSTRiP, genomic DNA PCR was performed. Primer pairs spanning the extracted CNV were used and each amplicon was visualized by gel electrophoresis. Four patterns of PCR product was detected: the deleted allele being larger or smaller compared to the prediction (case 1 and 2, respectively), absence of deleted allele (case 3) or non-deleted allele (case 4). In case 4, PCR was carried out again with primer pairs which amplify overhanging region between CNV and its outer region. Red and black lines in the diagram representing gel images indicate deleted and non-deleted allele, respectively. [file 1471-2164-15-240-S19.PDF]

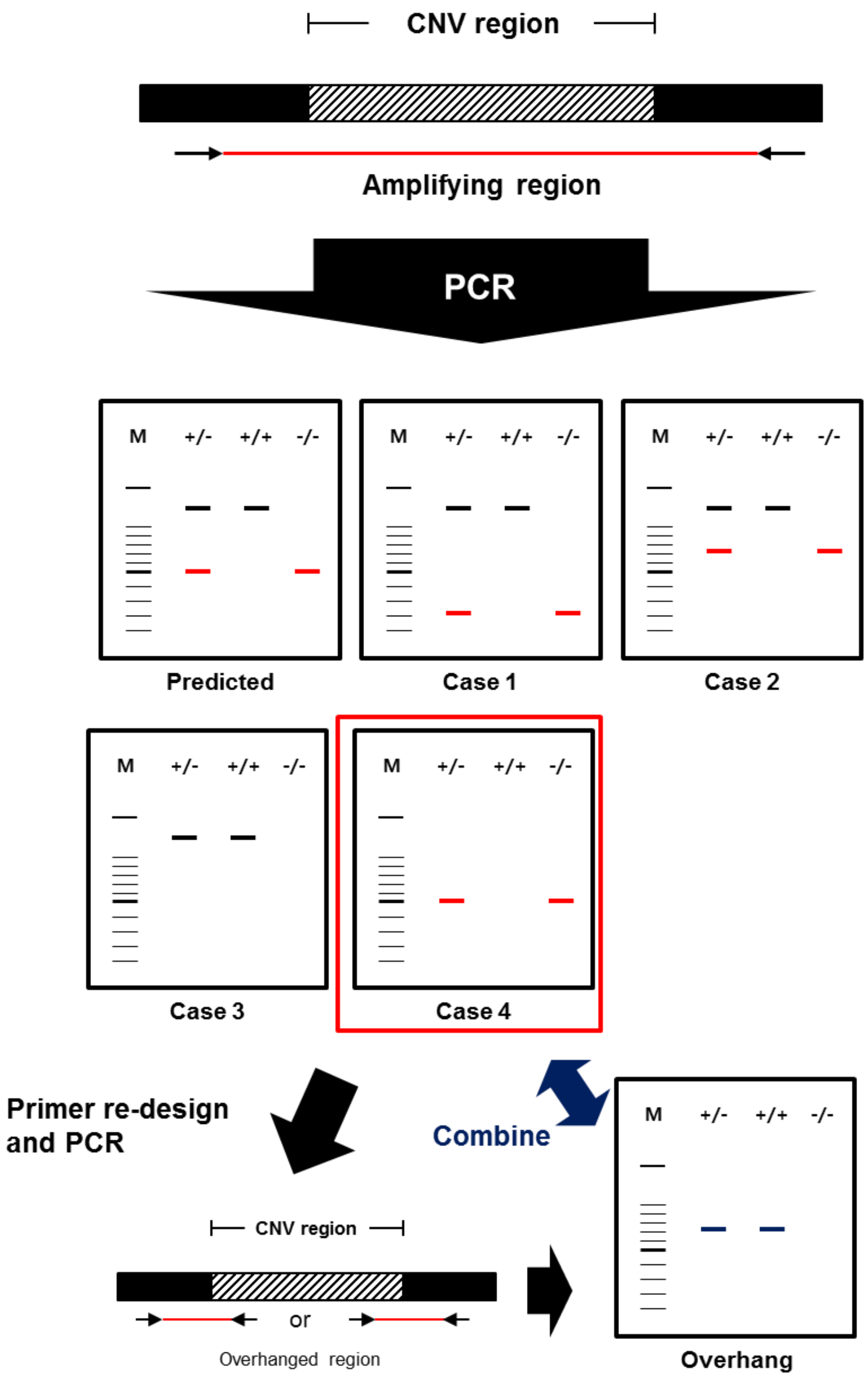

CNV region
Amplifying region
PCR
M +/- +/+ -/-
Predicted
M +/- +/+ -/-
Case 1
M +/- +/+ -/-
Case 2
M +/- +/+ -/-
Case 3
M +/- +/+ -/-
Case 4
Primer re-design and PCR
Combine
M +/- +/+ -/-
CNV region
or
Overhanged region
Overhang
